# Cyclic di AMP phosphodiesterase nanovaccine elicits protective immunity against *Burkholderia cenocepacia* infection in mice

**DOI:** 10.1038/s41541-025-01074-4

**Published:** 2025-02-01

**Authors:** Wesam E. Gawad, Yosra I. Nagy, Tamer M. Samir, Ahmed Mohamed Ibrahim Mansour, Omneya M. Helmy

**Affiliations:** 1 Department of Microbiology and Immunology, College of Pharmaceutical Sciences and Drug Manufacturing, Misr University for Science and Technology, 6th of October City, Egypt; 2 Department of Microbiology and Immunology, Faculty of Pharmacy, Cairo University, Cairo, Egypt; 3 Department of Pharmacology and Toxicology, Faculty of Pharmacy (Boys), Al-Azhar University, Cairo, Egypt; 4 Department of Pharmacology and Toxicology, College of Pharmacy, University of Hilla, Babylon, Iraq

**Keywords:** Protein vaccines, Adjuvants

## Abstract

*Burkholderia cenocepacia* causes life-threatening infections in immunocompromised patients. Treatment is challenging due to intrinsic antibiotic multiresistance, so vaccination provides an alternative approach. We aimed to identify vaccine candidates using reverse vaccinology and evaluate their efficacy as protein-loaded chitosan: pectin nanoparticles (C:P NPs) in a vaccine model. Applying strict subtractive channels, three proteins were shortlisted: WP_006481710.1 (LY), WP_012493605.1 (KT), and WP_006492970.1 (BD). Proteins were cloned, purified as His-tagged proteins, and loaded onto C:P NPs. Vaccinated mice had significantly higher systemic IgG and mucosal IgA antibody responses and induced IL-6 and IL-17A. 6x-His-LY-CS:P NPs and 6x-His-KT-CS:P NPs vaccines induced TNF-α. Vaccines conferred significant protection against *B. cenocepacia* intranasal infections. In conclusion, cyclic-di-AMP phosphodiesterase (WP_012493605.1) is a promising vaccine candidate that elicited IgG and IgA antibodies, Th1, Th2, and Th17 cellular immunity in BALB/c mice and protected against *B. cenocepacia* infection. This provides hope for saving lives of people at high risk of infection.

## Introduction

*Burkholderia cepacia* complex (Bcc) is a group of Gram-negative, opportunistic beta-proteobacteria comprising at least 24 genetically distinct species^1,2^. Bcc infection is life-threatening, particularly for cystic fibrosis (CF) and chronic granulomatous disease (CGD) patients. It has recently become a significant pathogen in immunocompromised and hospitalized patients^3–6^. Additionally, cepacia syndrome, a fatal necrotizing pneumonia caused by pulmonary colonization with Bcc, often leads to early death due to septicemia^7^. Among the members of the Bcc, *B. cepacia, B. cenocepacia, B. multivorans, B. dolosa, B. stabilis, and B. vietnamensis* are the most prevalent species in CF infections worldwide^8^. *B. cenocepacia* infections are usually associated with a poorer prognosis, and exhibit higher transmissibility and mortality than other members^9^.

*B. cenocepacia* exhibits heterogeneous resistance to various antibacterial agents due to efflux pump overexpression, mutations in drug targets, reduced cell permeability, enzymatic inactivation (lactamases, aminoglycoside-inactivating enzymes, and dihydrofolate reductase), restrictive porins, outer membrane permeability barriers, alteration in drug targets, or antibiotic modification^10,11^. In addition, it can form biofilms that significantly enhance antibiotic resistance^12^. However, studies have shown that in CF patients, Bcc are primarily found as single cells or small clusters within phagocytes and mucus, rather than as “biofilm-like structures”^13^. Currently, no effective therapies are available to eradicate *B. cenocepacia* infections owing to its multiple virulence factors, besides the intrinsic and acquired resistance to various antimicrobial agents^10,14^. Therefore, developing novel strategies to protect against Bcc infections is urgent^15^.

Prophylactic vaccination is a valuable approach for protecting vulnerable people and limiting transmission and antibiotic resistance. Only a few studies have been performed to develop vaccines against *B. cenocepacia*^15^. *Burkholderia cenocepacia* is a respiratory pathogen that can survive intracellularly. Therefore, developing a vaccine that generates a mucosal secretory IgA response would be an effective approach to preventing bacterial colonization. When designing such a vaccine, it is important to consider the optimal balance between Th1 (cellular immune response) and Th2 (humoral immune response), as both are necessary for effective pathogen clearance^15,16^. Additionally, Th17 cells have been shown to enhance antibacterial mucosal defenses and probably mediate protective vaccine-induced responses^17^. Vaccine development using whole-cell organisms, either inactivated or live attenuated, is not preferred due to the unnecessary antigenic load that leads to non-specific immune responses^18^, culturing problems, time-consumption, inaccurate and variable products, insufficient attenuation, high cost, lower immunogenicity, and hypersensitivity of the antigens^19^. Recently, the availability of whole bacterial genomic sequences and various in silico tools has revolutionized the identification and screening of potential vaccine candidates through a computer-based approach called ‘Reverse Vaccinology’ (RV)^20–22^, overcoming many limitations of whole-cell vaccination^23^. The first example of successfully applying RV is the serogroup B *Neisseria meningitidis* (MenB) vaccine^24^. It was successfully applied to *Streptococcus pneumoniae*^25^*, Mycobacterium tuberculosis*^26^*, Staphylococcus aureus*^27^*, Bacillus anthracis, Burkholderia pseudomallei*^28^, and coronavirus COVID-19^29^.

Vaccines must be co-administered with penetration enhancers, adjuvants, or encapsulated in particles^30^. Chitosan is a natural polymer synthesized by the deacetylation of chitin. Chitosan nanoparticles have immunological activity and mucoadhesive properties and can be used as an adjuvant for vaccine delivery^31^. Using chitosan nanoparticles (CNPs) with intranasal influenza subunit vaccine enhanced the systemic and mucosal antibody and cell-mediated immune responses^32^. Similarly, intranasal *Shigella flexneri* recombinant antigen chitosan nanovaccine showed an increase in the levels of IgG and IgA^33^.

In this study, bioinformatics and immunoinformatic approaches were utilized to predict protective protein candidates in *B. cenocepacia* through mining different available databases using strict inclusion and exclusion criteria to yield potential safe vaccine candidates. The recombinant chitosan-loaded vaccines were tested in BALB/c mice; they elicited strong mucosal, humoral, and cell-mediated immune responses and protected against *B. cenocepacia* infection.

## Results

### Identification of potential vaccine candidates in *Burkholderia cenocepacia* J2315 by subtractive bio-immunoinformatic tools

In the current study, 6993 protein sequences of *B. cenocepacia* J2315 were retrieved in FASTA format from the UniProtKB database (http://www.uniprot.org) (accessed on the 4th of September 2021). In addition, the complete genome of *B. cenocepacia* strain J2315, including three circular chromosomes and one plasmid with accession numbers NC_011000.1, NC_011001.1, NC_011002.1, and NC_011003.1 were retrieved from the National Center for Biotechnology Information (NCBI) database.

**Fig. 1:**
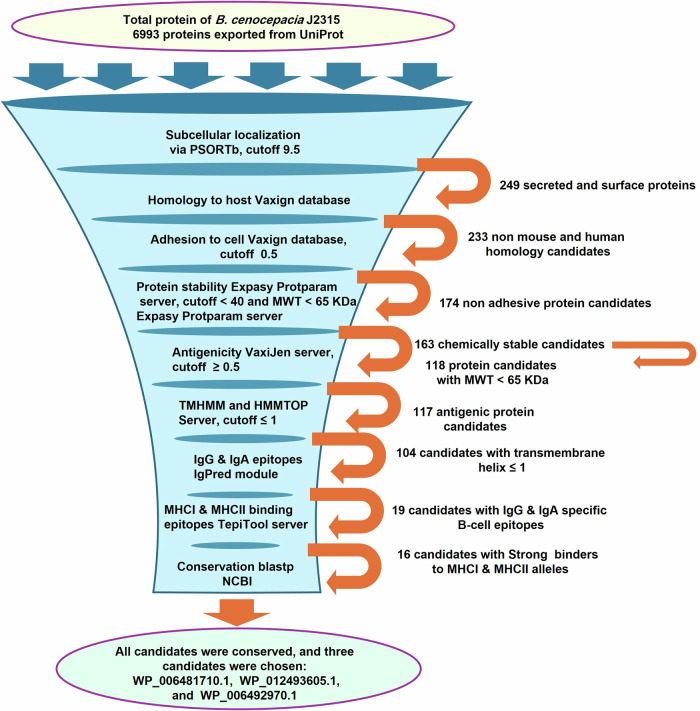
Schematic diagram representing the workflow of the bioinformatic analyses for predicting immunogenic targets in *B. cenocepacia* using the reverse vaccinology approach. Bioinformatics and immunoinformatic tools were used to analyze the *B. cenocepacia* proteome to identify potential vaccine candidates, where the proteins progressing to the next stage of analysis are written next to the orange arrow.

PSORTb analysis of *B. cenocepacia* J2315 proteome revealed the following distribution: 226 periplasmic, 95 extracellular, 154 outer membrane, 3073 cytoplasmic, 1494 cytoplasmic membrane proteins and 1951 proteins of unknown location (Fig. 1). Outer membrane and extracellular proteins (*n* = 249) were analyzed via the Vaxign system, where 233 proteins showed non-homology to human and mouse proteins, of which 174 had a probability of adhesion (Fig. 1). Computing the chemical stability, a measure of invitro degradation^34^, and molecular weight of the probably adhesive proteins using the Expasy ProtParam tool revealed 163 chemically stable proteins, of which 118 proteins had molecular weights less than 65 kDa (Fig. 1). The shortlisted candidates (*n* = 118) were screened for antigenicity via VaxiJen, where 117 proteins were predicted to be antigenic (Fig. 1). To predict the number of transmembrane helices and exclude proteins with more than one transmembrane helix proteins can be cloned and expressed efficiently, TMHMM v2.0 and HMMTOP 2.0 tools were used, by which 13 proteins were delisted (Fig. 1, Supplementary Table 1).

The shortlisted vaccine candidates (*n* = 104) were ranked based on the probability of containing immunogenic epitopes using the IgPred web server, where the number of antibody-specific (IgG and IgA) B cell epitopes present in each protein candidate was determined. Fifty protein candidates were delisted as they did not contain any B cell epitopes or had few IgG-specific B cell epitopes; 35 protein candidates were delisted as they did not contain or had few IgA-specific B cell epitopes; 19 protein candidates were shortlisted, as they had a high number of IgG (cutoff ≥25) and IgA (cutoff ≥6) specific B cell epitopes (Fig. 1, Supplementary Table 2). The TepiTool server was used to determine the number of MHC I and MHC II epitopes in the shortlisted protein candidates (*n* = 19) and their affinity to the chosen alleles. Accordingly, (*n* = 2) and (*n* = 1) proteins were delisted based on analysis of the number of MHC I and MHC II epitopes and their affinity for the binding alleles, respectively (Fig. 1, Supplementary Tables 3 and 4). To identify antigens providing cross-protection against Burkholderia species: *B. cenocepacia*, *B. multivorans*, *B. vietnamiensis, B. dolosa, B. contaminans, B. stabilis*, and *B. cepacia*, the shortlisted vaccine candidates (*n* = 16) were blasted using BLASTp against the most common Bcc species: *B. cenocepacia*, *B. multivorans*, *B. vietnamiensis, B.dolosa, B. contaminans, B. stabilis*, and *B. cepacia*. The alignment and Blast results revealed a high sequence similarity (% identity 85–100) with query coverage (80.4–100%) between the sequence of the blasted proteins and the tested species (Supplementary Table 5). Multiple sequence alignment of the complete sequence of the three shortlisted protein candidates, used in further experiments, (Table 1): LY (WP_006481710.1), KT (WP_012493605.1), and BD (WP_006492970.1) proteins with representative strains of the most prevalent Bcc species (top 20 sequences) was performed using Clustal Omega, and visualized using the online program NCBI multiple sequence alignment viewer (version 1.25.1, NCBI, MD, USA) (Supplementary Fig. 1).

**Table 1:** Putative vaccine candidates used in the vaccination model evaluated in terms of subcellular localization, antigenicity, adhesion, number of transmembrane helices, and number of IgG and IgA epitopes through the Psortb, VaxiJen, Vaxign, and TMHMM v2.0, HMMTOP 2.0., and Igperd

Protein code	Protein Uniprot entry	Accession number	Protein annotation	Localization by Psortb	Similarity to human and mouse	Stability prediction (ProtParam threshold ≤ 40)	Adhesion probability	Antigenicity score threshold > 0.5	Number of Transmembrane helices	Number of IgG epitopes	Number of IgA epitopes
LY	B4EJ27	**WP_006481710.1**	Phospholipase C	Extracellular	None	28.58	0.875	0.6678	0	39	22
KT	B4EKU7	**WP_012493605.1**	Phosphodiesterase superfamily protein AtaC domain	Extracellular	None	20.64	0.702	0.6074	0	47	20
BD	B4EJA5	**WP_006492970.1**	Chitinase class I domain	Extracellular	None	25.12	0.919	1.0521	0	27	9

**Fig. 2:**
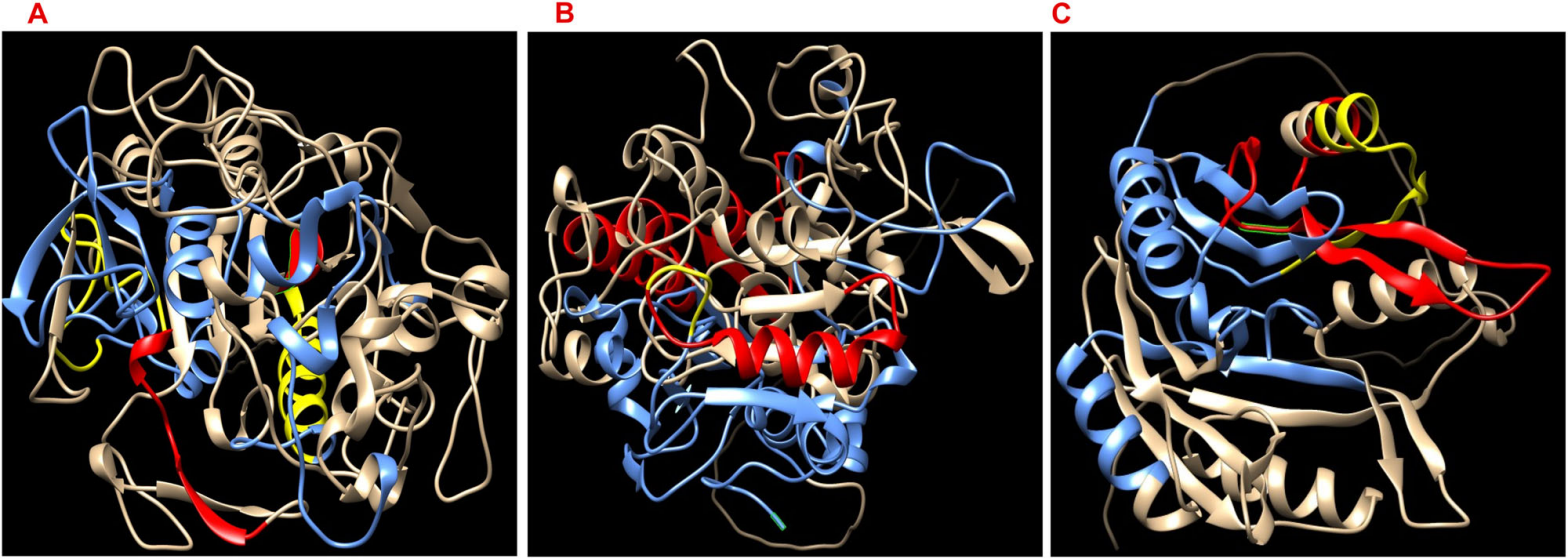
Predicted 3D structure of LY, KT, and BD proteins using Swiss model and visualization using Chimera. The predicted 3D structures of LY (**A**), KT (**B**), and BD (**C**) show the exposed B cell epitopes on the protein surface highlighted blue for IgG, red for IgA, and yellow for both IgG and IgA epitopes.

Homology modeling and prediction of the three-dimensional (3D) structure of LY, KT, and BD proteins was conducted using the Swiss model. Surface exposure of B cell epitopes, including IgG and IgA, was highlighted and visualized via Chimera (Fig. 2). The homology model confirmed that the three selected proteins have surface-exposed epitopes freely accessible to bind with antibodies.

### Successful cloning and expression of 6x-His-LY, 6x-His-KT, and 6x-His-BD proteins

The DNA fragments encoding WP_006481710.1 (LY), WP_012493605.1 (KT), and WP_006492970.1 (BD) proteins were PCR amplified using the genomic DNA of the *B. cenocepacia* J2315. The resulting amplicons of sizes 1671 bp, 1644 bp, and 1005 bp were cloned into the pET22b (+) vector (Supplementary Fig. 2), and *E. coli* Top 10 was transformed by the recombinant plasmids (pET22b-LY, pET22b-KT, and pET22b-BD). Successful clones were confirmed by PCR (Supplementary Figs. 2 and 3).

**Fig. 3:**
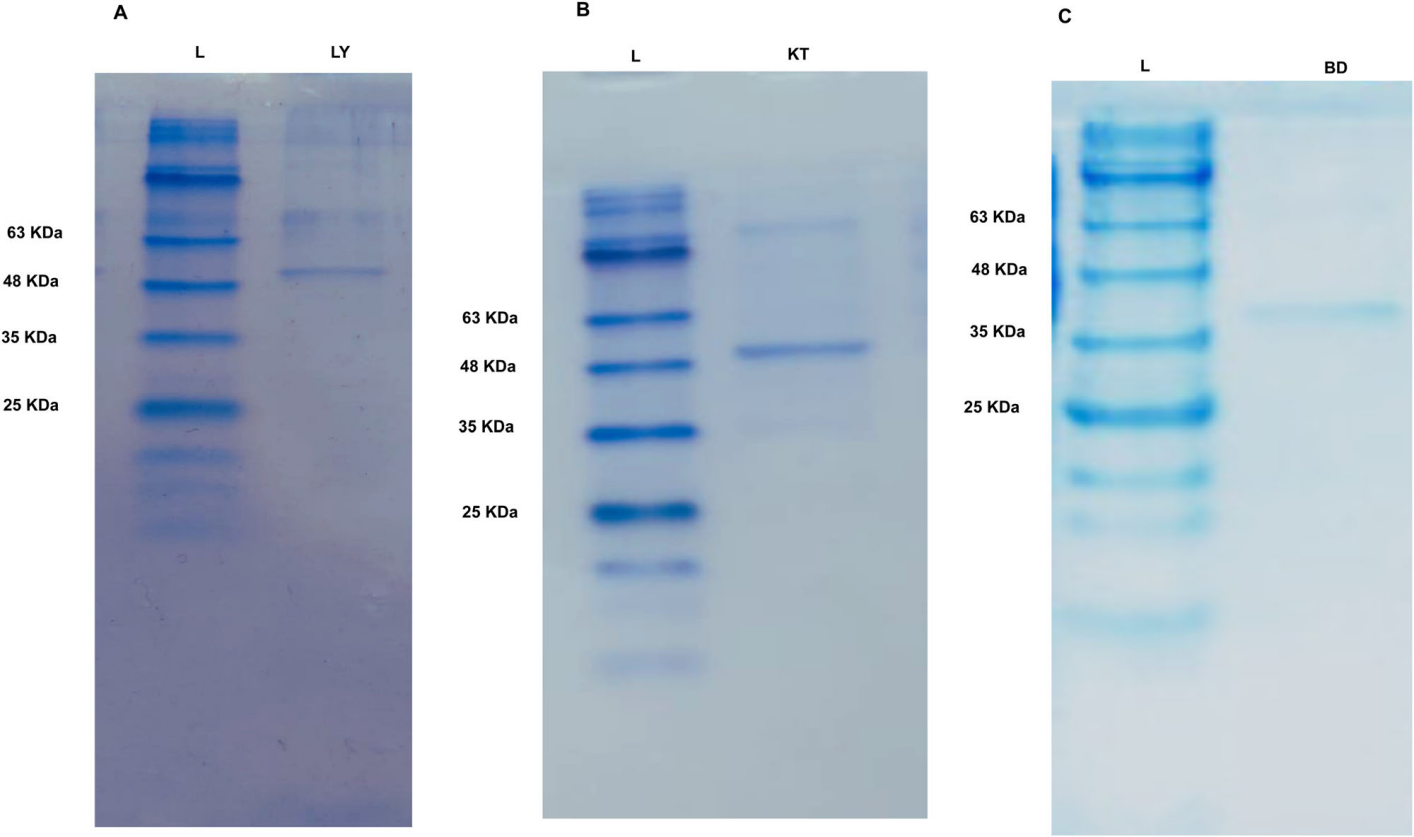
Purification of recombinant His-tagged proteins 6x-His-LY, 6x-His-KT, and 6x-His-BD using Ni-NTA columns. A photograph of coomassie blue-stained SDS-PAGE gel of Ni-NTA purified recombinant: **A** 6x-His-LY with a size of approximately 59.2 KDa, **B** 6x-His-KT with a size of approximately 57.9 KDa, and **C** 6x-His-BD with a size of approximately 35.1 KDa.

The cloned 6x-His-LY, 6x-His-KT, and 6x-His-BD proteins comprised 557 amino acids, 548 amino acids, and 335 amino acids, besides the six amino acids of the His-tag; the whole fragments were predicted to have a size of 59.2 kDa, 57.89 kDa, and 35.08 kDa, using (Bioinformatics.Org/sms/prot_mw.html). *E. coli* BL21 (DE3) was transformed by the recombinant plasmids, pET22b- LY, pET22b-KT, and pET22b- BD, and the 6x-His-tagged proteins were purified using Ni-NTA columns. Protein bands with the expected sizes were visualized on SDS-PAGE (Fig. 3, Supplementary Fig. 4).

### Characterization of the prepared recombinant proteins loaded chitosan-pectin nanoparticles

**Fig. 4:**
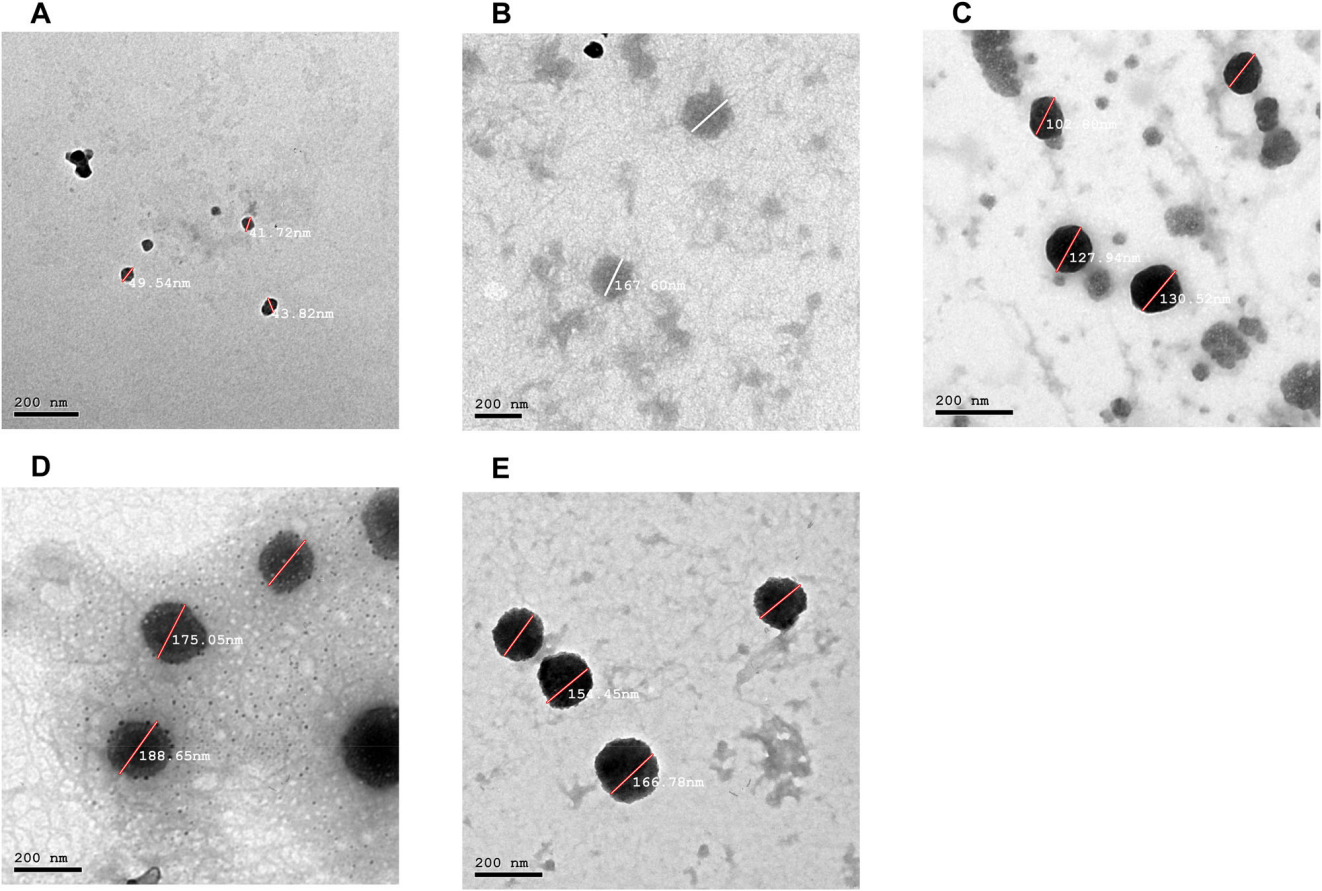
The recombinant 6x-His-tagged proteins were successfully loaded onto chitosan-pectin nanoparticles. The morphological characterization of the nanoparticles was evaluated by Transmission Electron Microscopy. Micrographs of the prepared: **A** CS:P NPs, **B** Non-specific antigen control BSA-CS:P NPs, **C** 6x-His-LY-CS:P NPs, **D** 6x-His-KT-CS:P NPs, and **E** 6x-His-BD-CS:P NPs were taken using a JEOL 2010 Transmission Electron Microscope. Bar indicates 200 nm. CS:P chitosan-pectin, BSA Bovine serum albumin.

The chitosan-pectin nanoparticles (CS:P NPs) were prepared and loaded with the purified 6x-His-LY, 6x-His-KT, and 6x-His-BD proteins. The percentage of loaded proteins was determined by Bradford assay and was equivalent to 71.5%, 78%, and 84%, respectively. Chitosan-pectin nanoparticles loaded with bovine serum albumin (BSA) served as non-specific antigen control. Morphology and size of the prepared CS:P NPs, CS:P-BSA NPs, 6x-His-LY-CS:P NPs, 6x-His-KT-CS:P NPs, and 6x-His-BD-CS:P NPs were determined by transmission electron microscopy (TEM). The prepared NPs were spherical (Fig. 4). The characteristics of the prepared CS:P NPs, 6x-His-LY-CS:P NPs, 6x-His-KT-CS:P NPs, and 6x-His-BD-CS:P NPs are summarized in Table 2, where values recorded are the mean ± SD for the size, polydispersity index and zeta potential.

**Table 2:** Physical-Structural Characterization of the prepared CS:P NPs, 6x-His-LY-CS:P NPs, 6x-His-KT-CS:P NPs, and 6x-His-BD-CS:P NPs

Nanoparticles	Mean of particle Size (nm)	Polydispersity index	Zeta Potential (mV)	Loading efficiency (%)
CS:P NPs	202 ± 1.09	0.437 ± 0.04	21.7 ± 1.3	-
6x-His-LY-CS:P NPs	287 ± 43.5	0.532 ± 0.01	32 ± 2.5	71.5%
6x-His-KT-CS:P NPs	212 ± 8.3	0.448 ± 0.04	12.2 ± 1.04	78%
6x-His-BD-CS:P NPs	234.2 ± 14.7	0.468 ± 0.02	32.4 ± 5.4	84%

### Successful lung inflammation in BALB/c mice following intranasal infection by *B. cenocepacia*

**Fig. 5:**
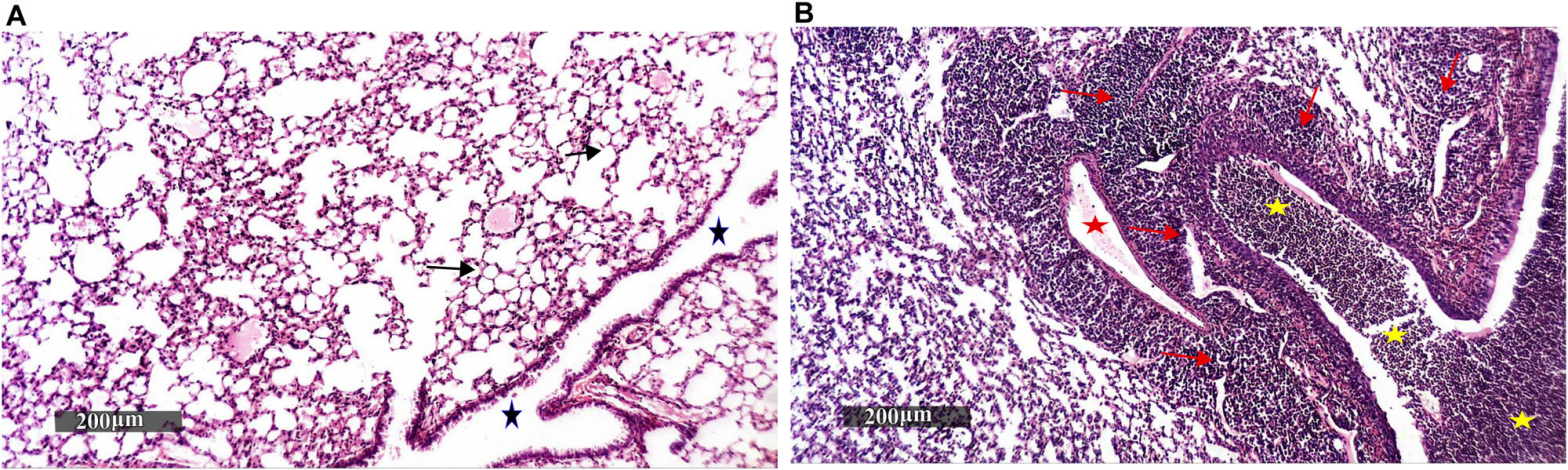
Lung inflammation in mice infected with *B. cenocepacia*. Paraffin lung sections from three mice infected with *B. cenocepacia*, five days post-intranasal infection, and three healthy mice were stained with Harris Hematoxylin and Eosin and observed under a microscope with a magnification power of 200×, where: **A** photograph showing lung tissue sections of healthy mice with well-organized histological features of pulmonary parenchyma with thin-walled interalveolar septa and normal vasculatures (arrow), intact alveolar luminal spaces, intact bronchiolar epithelium as well as minimal records of mononuclear inflammatory cells infiltrates in peribronchiolar tissue with intact intraluminal spaces (star), **B** photograph showing lung tissue sections of infected mice with severe bronchiolitis and remarkable intraluminal cellular debris with mixed inflammatory cells (yellow star), severe interstitial pneumonia with a marked increase of peribronchiolar mixed inflammatory cells infiltrates (red arrow) that extend to alveolar spaces, and congested vasculatures (red star).

All mice showed 100% survival without any signs of distress. The successful pulmonary infection with *B. cenocepacia*, five days following intranasal infection, was confirmed by retrieval of 4 × 10^5^ CFU/mL of *B. cenocepacia* from the lungs of infected mice, and a comparative histopathological examination of lungs from infected and healthy mice. Harris Hematoxylin and Eosin (H&E) stained lung sections revealed that the lung tissue of infected mice was characterized by severe bronchiolitis with remarkable intraluminal cellular debris with mixed inflammatory cells, severe interstitial pneumonia with a marked increase of peribronchiolar mixed inflammatory cell infiltrates that extend to alveolar spaces, and congested vasculatures compared to healthy mice (Fig. 5).

### Recombinant proteins loaded chitosan-pectin nanoparticles trigger systemic and mucosal immune responses in BALB/c mice

**Fig. 6:**
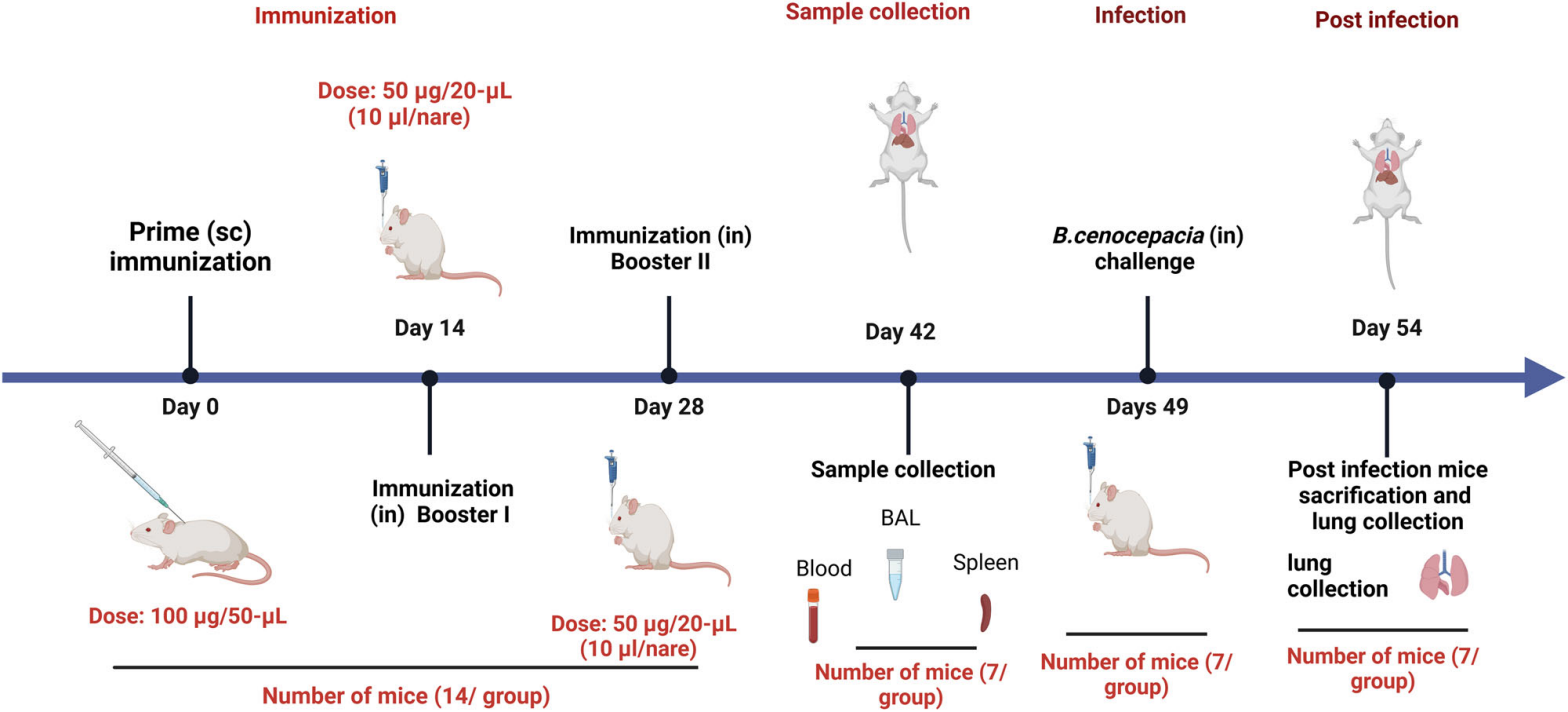
The timeline of immunization and overall in vivo work through the terminal point on day 54. Schematic illustration of mice immunization, sample collection, and *B. cenocepacia* challenge is shown. Five groups of female BALB/c mice (14 mice/group) were subcutaneously (sc) prime-vaccinated with 6x-His-LY-CS:P NPs, 6x-His-KT-CS:P NPs, or 6x-His-BD-CS:P NPs, and with PBS or adjuvant as their respective controls. Mice (*n* = 14/group) were intranasally (in) boosted twice on days 14 and 28. On day 42, sera, bronchoalveolar lavage (BAL), and spleens were collected from 7 mice/ group. On day 49, the remaining mice (*n* = 7/group) were infected intranasally with approximately 1 × 10^8^ colony-forming units (CFUs) of *B. cenocepacia*. Five days post-infection, lungs were harvested, homogenized, serially diluted, and spread on LB agar plates to determine the bacterial load. The diagram is generated using BioRender (license number *JI27JQG39D*).

**Fig. 7:**
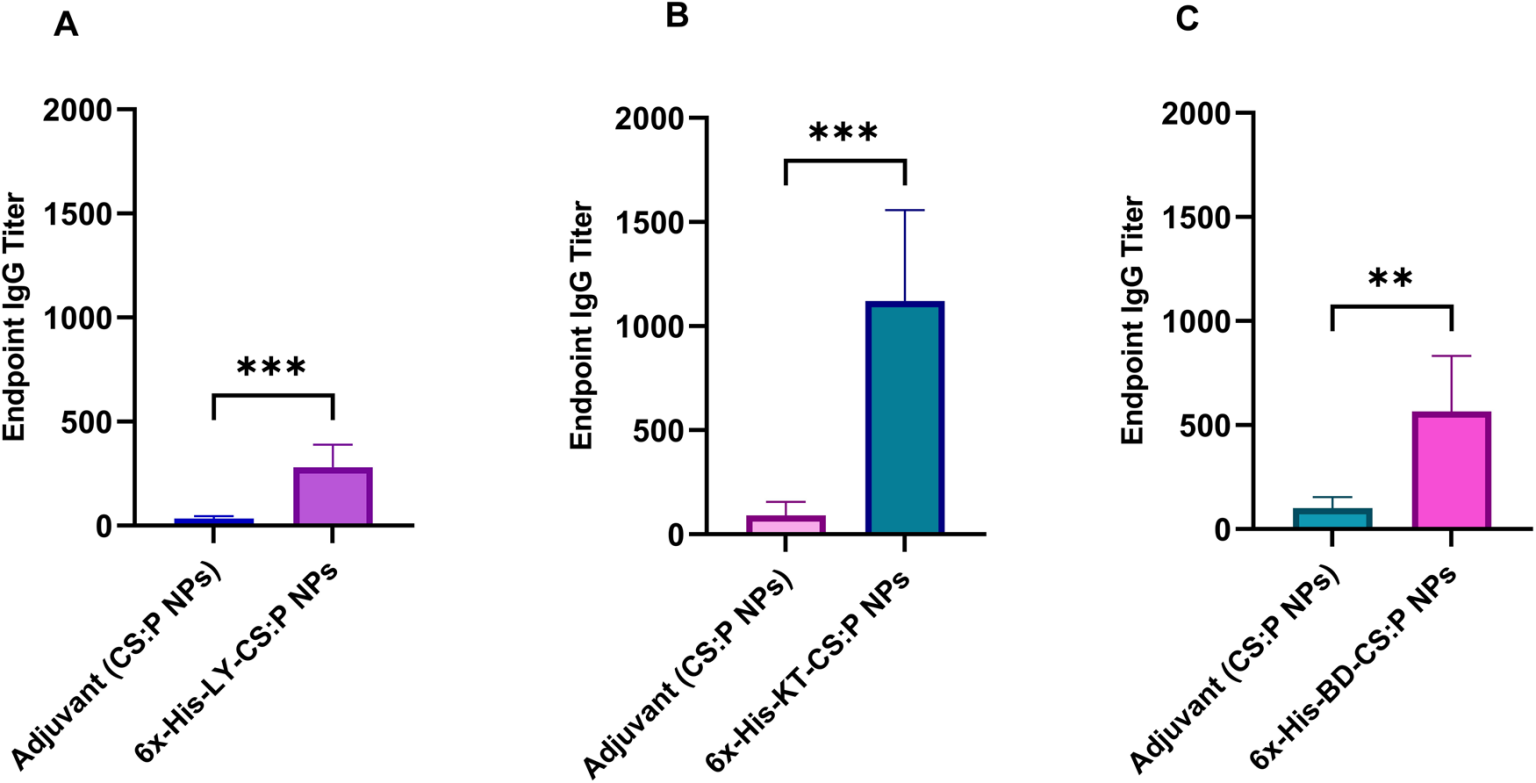
6x-His-LY-CS:P NPs, 6x-His-KT-CS:P NPs, and 6x-His-BD-CS:P NPs induce systemic humoral immune responses in BALB/c mice. Mice were immunized subcutaneously with a prime dose and two booster intranasal doses of **A** 6x-His-LY-CS:P NPs, **B** 6x-His-KT-CS:P NPs, **C** 6x-His-BD-CS:P NPs, and adjuvant (CS:P). Two weeks following the last dose, mice (*n* = 7 per group) were sacrificed, sera collected, and the IgG antibodies were detected using indirect ELISA. The Y-axis represents the endpoint titers; the error bars indicate S.D. The unpaired *t*-test was used to determine the significance of the antibody titer; the asterisk refers to statistically significant differences as follows: (**) *p*-value < 0.01, and (***) *p*-value < 0.001.

**Fig. 8:**
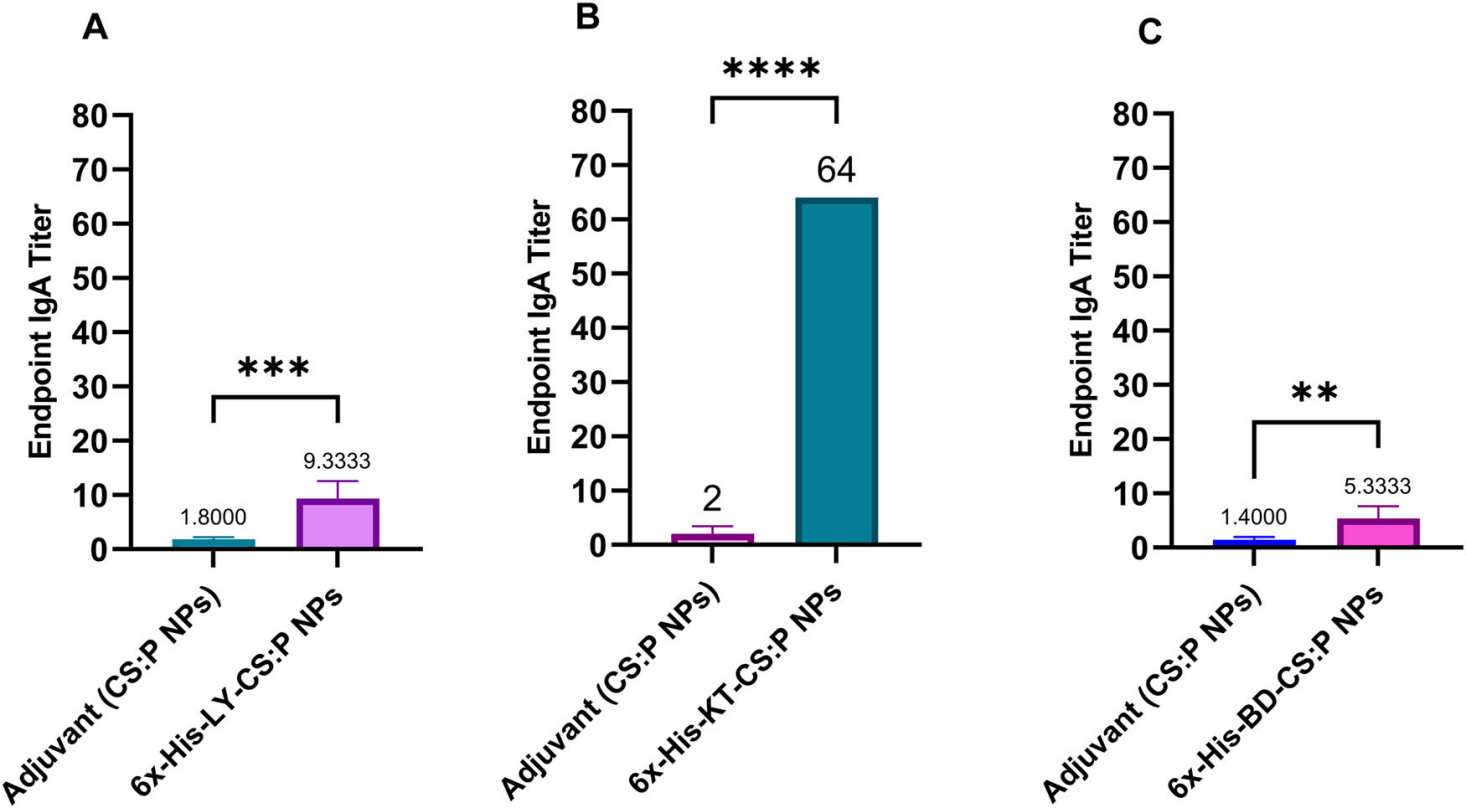
6x-His-LY-CS:P NPs, 6x-His-KT-CS:P NPs, and 6x-His-BD-CS:P NPs induce mucosal immune response in the respiratory tract of BALB/c mice. Mice were immunized subcutaneously with a prime dose and two booster intranasal doses of **A** 6x-His-LY-CS:P NPs, **B** 6x-His-LY-CS:P NPs, **C** 6x-His-LY-CS:P NPs, adjuvant (CS:P). Two weeks following the last dose, mice (*n* = 7 per group) were sacrificed, the bronchoalveolar lavage was collected, and the IgA antibodies were detected using indirect ELISA. The results were expressed as the endpoint titer; error bars indicate S.D. The unpaired t-test was used to determine the significance of the antibody titer; asterisk refers to statistically significant differences as follows: (**) *p*-value < 0.01, (***) *p*-value < 0.001, and (****) *p*-value < 0.0001.

The potential of the 6x-His-LY, 6x-His-KT, and 6x-His-BD loaded CS:P NPs to elicit serum and mucosal antibody-mediated immune responses in BALB/c mice following a subcutaneous prime and two intranasal booster doses was evaluated two weeks after the last booster dose (Fig. 6), using indirect ELISA. The serum IgG titers of vaccinated mice with any of the tested vaccine candidates were significantly higher than mice receiving CS:P NPs (*p-*value < 0.01); 6x-His-KT and 6x-His-LY vaccinated mice had the highest IgG responses (Fig. 7). The IgA titers in bronchoalveolar lavage (BAL) of vaccinated mice with any of the tested vaccine candidates were significantly higher than mice receiving CS:P NPs (*p-*value < 0.05); 6x-His-KT vaccinated mice had the highest IgA response (Fig. 8).

### Recombinant proteins loaded chitosan-pectin nanoparticles induce cellular immune response in BALB/c mice

**Fig. 9:**
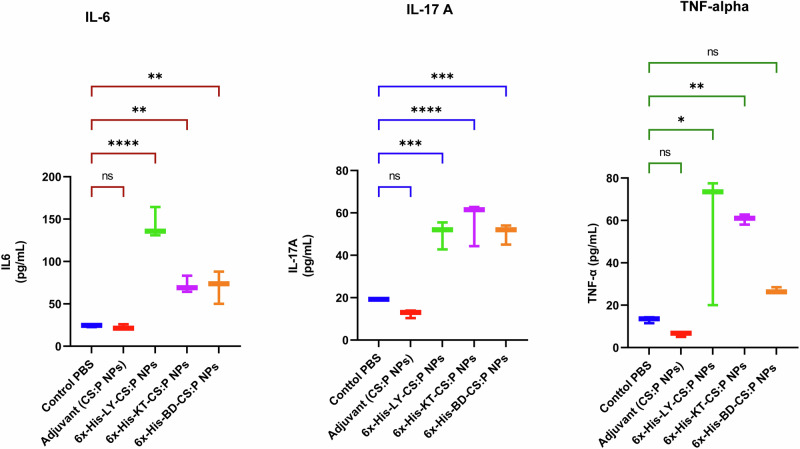
Immunization with 6x-His-LY-CS:P NPs, 6x-His-KT-CS:P NPs, and 6x-His-BD-CS:P NPs elicits cellular immune response in vaccinated mice. Mice were immunized subcutaneously with a prime dose and two booster intranasal doses of 6x-His-LY-CS:P NPs, 6x-His-KT-CS:P NPs, and 6x-His-BD-CS:P NPs. Two weeks following the second booster dose, the spleens from three animals from each group were aseptically dissected, cultured, and restimulated with the respective vaccine for 72 h. Interleukin-6 (IL-6), interleukin 17 A (IL-17A), and tumor necrosis factor-alpha subunit (TNF-α) production in cell supernatants was measured using ELISA. The data were analyzed using one-way ANOVA followed by Dunnett’s multiple comparisons test. Box and whiskers plot (min to max), and the horizontal bars represent means ± standard deviation (SD). Asterisk refers to statistically significant differences as follows: (*) *p*-value *<* 0.05, (**) *p*-value *<* 0.01, (***) *p*-value *<* 0.001, and (****) *p*-value *<* 0.0001, ‘ns’ for non-significance.

To evaluate the cytokine response profile towards the tested vaccine candidates, the spleens of immunized mice were dissected two weeks following the second booster dose (Fig. 6), cultured, in the presence of 6x-His-LY-CS:P, 6x-His-KT-CS:P, or 6x-His-BD-CS:P nanoparticles for 72 h. Interleukin-6 (IL-6), interleukin 17 A (IL-17A), and tumor necrosis factor-alpha subunit (TNF-α) levels were measured in the cell-free supernatant using quantitative ELISA. The levels of IL-6 and IL-17A were significantly higher in immunized mice compared to the control groups receiving either PBS or CS:P NPs (*p-*value < 0.05). Significant activation of TNF-α was also observed in groups receiving 6x-His-LY-CS:P NPs and 6x-His-KT-CS:P NPs (*p-*value < 0.05); however, no significant activation of TNF-α was detected in 6x-His-BD-CS:P NPs group compared to the phosphate-buffered saline (PBS) control group. 6x-His-KT-CS:P NPs induced the highest level of IL-17A and TNF-α, while 6x-His-LY-CS:P NPs induced the highest level of IL-6 (Fig. 9).

### Serum of immunized mice has a bactericidal activity against *B. cenocepacia*

**Fig. 10:**
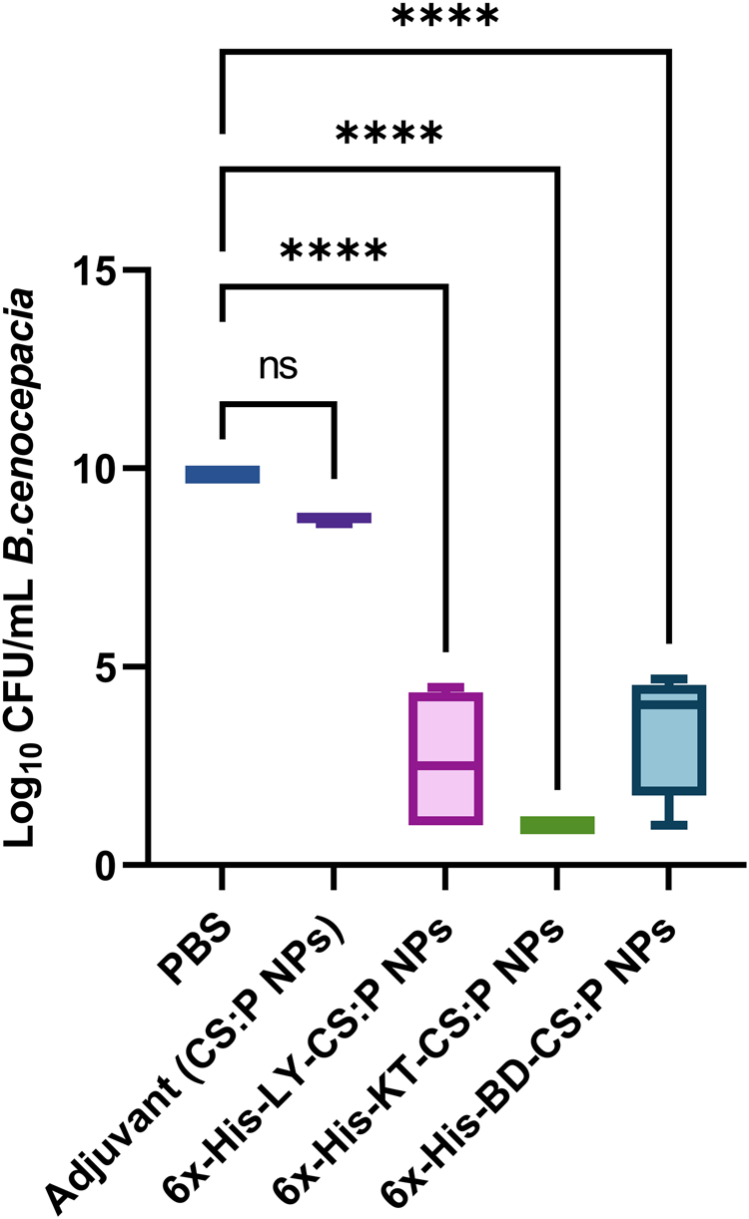
Bactericidal activity of vaccinated mice sera against *B. cenocepacia*. Mice were immunized subcutaneously with a prime dose and two booster intranasal doses of 6x-His-LY-CS:P NPs, 6x-His-KT-CS:P NPs, 6x-His-BD-CS:P NPs, adjuvant (CS:P), and phosphate-buffered (PBS) saline. Two weeks following the last dose, mice (*n* *=* 7 per group) were sacrificed, and the sera were collected, pooled, and incubated with 50 μL *B. cenocepacia* J2315 cultures (3 × 10^4^ CFU). The Y-axis represents log_10_ CFU/mL *B. cenocepacia* with a minimum load of detection of 10 CFU. Box and whiskers plot (min to max), and the horizontal bar represents the min to max of the log_10_ CFU/mL count, and error bars indicate S.D. Statistical difference was determined using one-way ANOVA followed by Dunnett’s multiple comparisons test; Asterisk refers to statistically significant differences as follows: (****) *p*-value *<* 0.0001, ‘ns’ for non-significance.

The bactericidal activity of the sera obtained from immunized mice, two weeks following the last booster dose, was evaluated against *B. cenocepacia* using a colony reduction assay. The undiluted sera of vaccinated mice, with 6x-His-LY-CS:P NPs, 6x-His-KT-CS:P NPs, or 6x-His-BD-CS:P NPs had neutralizing antibodies capable of reducing the *B. cenocepacia* colony counts. A significant fold reduction (*p-*value < 0.05) in *B. cenocepacia* counts was detected in vaccinated mice, using sera from naïve mice as a baseline, and compared to sera of mice receiving CS:P NPs (Fig. 10).

### Enhanced pulmonary clearance in vaccinated mice challenged by intranasal *B. cenocepacia*

**Fig. 11:**
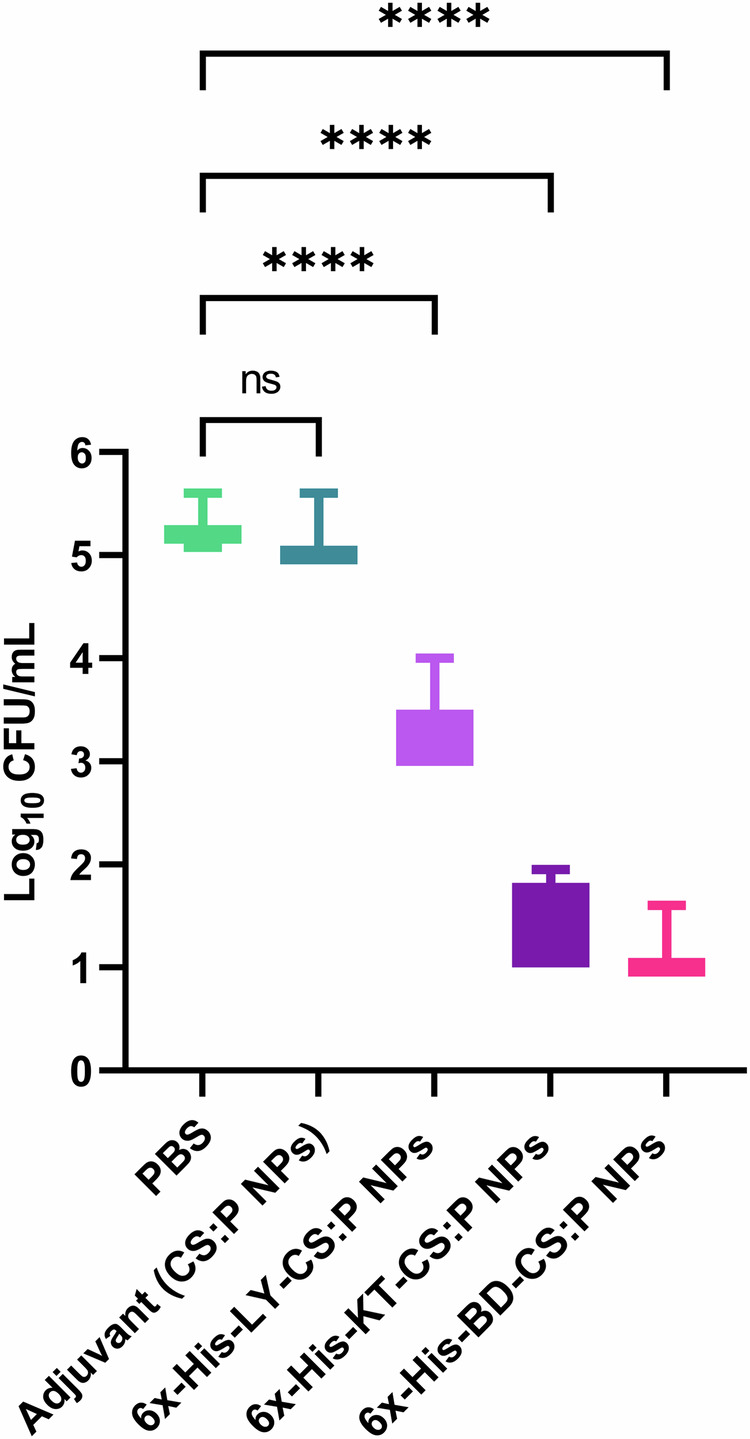
Immunization with 6x-His-LY-CS:P NPs, 6x-His-KT-CS:P NPs, and 6x-His-BD-CS:P NPs efficiently enhances *B. cenocepacia* pulmonary clearance. Colonization studies were performed three weeks following the last booster dose of 6x-His-LY-CS:P NPs, 6x-His-KT-CS:P NPs, 6x-His-BD-CS:P NPs, adjuvant, and phosphate- buffered saline. Vaccinated mice (*n* = 7/group) were infected intranasally with approximately 1 × 10^8^ colony-forming units (CFU) of *B. cenocepacia*. Five days following infection, lungs were harvested, homogenized, serially diluted, and spread on LB agar plates to determine the bacterial load with a minimum load of detection of 10 CFU. Box and whiskers plot (min to max), the horizontal bar represents the mean of the log_10_ CFU/mL count, and the error bars indicate S.D. Statistical difference was determined using one-way ANOVA followed by Dunnett’s multiple comparisons test; Asterisk refers to statistically significant differences as follows: (****) *p*-value < 0.0001, ‘ns’ for non-significance.

The protective efficacy of the proposed vaccine candidates against *B. cenocepacia* was evaluated using the pulmonary clearance model, in which all groups were challenged with *B. cenocepacia* via the intranasal route three weeks following the second booster dose (Fig. 6). 100% survival of mice without signs of distress was observed. Results revealed that the bacterial burden recovered from the lungs of vaccinated mice was significantly reduced (*p*-value ˂ 0.05) compared to those receiving either CS:P NPs or PBS. The 6x-His-LY-CS:P NPs, 6x-His-KT-CS:P NPs, or 6x-His-BD-CS:P NPs vaccinated mice showed significant log_10_ reduction in the *B. cenocepacia* counts in lungs, compared to the unvaccinated control group (PBS) (Fig. 11).

## Discussion

Bcc pneumonia infections in CF patients are accompanied by high morbidity and mortality rates owing to the disease’s invasiveness and the rapid decline in lung function^35,36^. *B. multivorans* and *B. cenocepacia* are the primary Bcc isolates from CF centers^6,37^. Meanwhile, outbreaks have also been reported in non-CF patients, including dialysis patients^38^, patients with indwelling medical devices^39^, intensive care patients^39^, and cancer patients^40^. In the current study, we employed a reverse vaccinology approach to screen immunogenic proteins using publicly available bioinformatic programs, thus saving the time and cost spent in conventional screening of vaccine candidates^21^. We cloned the shortlisted proteins and used them in a vaccination model.

Our strategy used strict subtractive approaches to initially screen target antigens based on their subcellular localization, unrelatedness with human and mouse proteomes, adhesion probability, chemical stability, and antigenicity^41^. Surface proteins, either secretory or part of the outer membrane, are typically the first pathogen proteins that encounter the host immune system and stimulate the immune responses^42–44^. Such proteins are imperative for pathogenicity, adhesion, and invasion, making them ideal vaccine candidates^45^. Non-homology to host proteins guards against an auto-immune response^46^. Adhesive proteins can initiate host cell responses and mediate bacterial entry rather than non-adhesive ones^18,47,48^.

The antigenicity of a protein is its ability to elicit immunogenic responses in the form of B cell or T-cell responses^49,50^. The chemical stability of proteins is an important physiochemical property in vaccine design. The instability index default was set to be less than 40 to avoid unstable proteins and provide the desired stability for initiating an immunogenic response^29,51–53^. Besides, molecular weight (MWT) was set to be less than 65 KDa, as proteins with a molecular weight higher than 100 kDa are difficult to produce in *E.coli*^18,52–54^.

Another employed criterion was selecting proteins with utmost one transmembrane helix, as such proteins can be cloned and expressed efficiently^18^. Membrane proteins, with their hydrophobic and hydrophilic surfaces, are difficult to solubilize and purify in their native conformations^55^. Our shortlisted candidates had no predicted transmembrane helices as recommended by previous studies^46,56^.

Antibodies exhibit an important role in humoral immunity, as B-cells recognize epitopes and produce antigen-specific antibodies^57^. The IgPred server predicts B cell epitopes with a fixed length of 20 amino acid residues at a threshold ≥0.9, helping to identify the ability to generate systemic (IgG) and mucosal (IgA) antibodies^58–61^. Whole protein-based vaccines could have a mix of high-affinity and low-affinity random epitopes. In this regard, selecting strong binders (high-affinity epitopes) for vaccine design is pivotal to inducing adequate inflammatory and immune responses. In the current study, the TepiTool server, a component of the IEDB, was used for the prediction of strong binders (percentile rank ≤1) for the 27 most frequent human MHC I alleles with 9-mer amino acid length and the prediction of strong binders (percentile rank ≤10) for 26 human MHC II alleles with 15-mer amino acid^62,63^. The frequency of alleles is critical for understanding the spread and pathogenicity of diseases^64^. The tested MHC I and MHC II binding alleles covered over 95% of the global population^62^.

To develop an efficient vaccine against different species of the Bcc, the shortlisted proteins were blasted against the most common Bcc species: *B. cenocepacia*, *B. multivorans*, *B. vietnamiensis*, *B. cepacia*, *B. contaminans*, *B. stabilis*, and *B. dolosa* proteomes using the NCBI BLASTp tool^65^. Highly conserved proteins having a pairwise identity of at least 80% and minimum query coverage of 80% across several Bcc species were selected^46^. Caro-Gomeza and coworkers considered proteins with identity and query coverage ≥60% as conserved proteins^66^, while Meunier and coworkers considered proteins with over 80% identity and 50% query coverage as conserved proteins^67^.

The NCBI conserved domain database^68^ was accessed to predict functional annotations for the proteins LY, KT, and BD. The phosphoesterase family protein (LY) is related to phospholipase C superfamily (PLC superfamily) COG3511, which catalyzes the hydrolysis of phosphatidylcholine to form 1,2-diacyl-sn-glycerol and phosphocholine^69^. PLC enzymes play important roles in the pathogenesis of bacterial infections^69^, including those caused by *Clostridium perfringens*, *Pseudomonas aeruginosa*^70^*, Mycobacterium tuberculosis*^71^*, Listeria monocytogenes*, and *Burkholderia pseudomallei*^69^. PLC is also present in CF pathogens, such as *P. aeruginosa* and *B. cenocepacia*^72^. *Mycobacterium abscessus* phospholipase C (MA-PLC) was tested as a vaccine candidate in CF mice, and it enhanced the immune response against *M. abcessus*^73^. The exported protein (KT) is conserved in the phosphodiesterase superfamily protein AtaC domain COG1524, which degrades cyclic-di-AMP. AtaC protein is conserved in microorganisms, such as *Streptococcus pneumoniae* and *Mycobacterium tuberculosis*^74^. When used as a vaccine in mice, the c-di-AMP phosphodiesterase (CnpB) recombinant protein elicited high levels of humoral response and lung mucosal immunity against *M. tuberculosis*^75^. We used the PSI-BLAST analysis of the NCBI non-redundant database to annotate the uncharacterized lipoprotein (BD) and this revealed a percentage identity of 59.75% with *Ralstonia mannitolilytica* protein accession number CAJ0710589 (query coverage of 94%); this protein is conserved in chitinase class I domain COG3179 accession cl46694. A previous study used RV to predict an uncharacterized secreted protein with a chitinase domain (PA0629) as a vaccine candidate in *Pseudomonas aeruginosa*^43^. In addition, a recombinant chitinase 3 protein showed promising activity as a vaccine against *Candida albicans*^76^.

Bcc colonization occurs in the respiratory mucosa, a vaccine that induces a mucosal immune response would be advantageous^16^. Recombinant proteins require effective adjuvants to elicit a protective mucosal immune response^33,77^. The purified 6x-His-LY, 6x-His-KT, and 6x-His-BD antigens were loaded on low-molecular-weight chitosan for nasal administration. Chitosan is an efficient adjuvant as it can adhere to the mucosal surface and deliver immunogenic antigens to immune cells^78,79^. Pectin is a natural polysaccharide used in food and pharmaceutical industries owing to its biodegradability^80^. Pectin is a useful drug carrier due to its mucoadhesive properties, which attach easily to mucosal surfaces^80^. Chitosan nanoparticles were used as a delivery system for the recombinant MxiH antigen of *Shigella flexneri*^33^, Hepatitis B^81^, swine influenza A virus^82^, and *Clostridioides difficile* vaccines^83^.

Particle size and surface charges of NPs play an important role in nanovaccine design^33,84^. Chitosan and trimethyl chitosan NPs with positive charges and sizes less than 500 nm are suitable for vaccination^33,85^. The prepared 6x-His-LY-CS:P NPs, 6x-His-KT-CS:P NPs, and 6x-His-BD-CS:P NPs had uniform particle sizes of less than 300 nm without aggregation, making them suitable for intranasal administration. NPs with particle sizes of approximately 300 or less are expected to have high cellular uptake^86^ and are easily captured by dendritic cells^87^. Another important factor to consider when preparing chitosan NPs is the zeta potential, which can affect particle stability and adhesion^85,88^. In the current study, a decrease in the zeta potential value from 21.7 in the control-free CS:P NPs to 12.2 after loading with 6x-His-KT-CS:P NPs was observed; however, an increase in the zeta potential value to 32 and 32.4 was recorded upon loading with 6x-His-LY-CS:P NPs and 6x-His-BD-CS:P NPs. The loading efficacy of the prepared recombinant His-tagged proteins on CS:P NPs ranged from 71.5 to 84%, which is comparable to loading efficiencies previously reported using the ionic gelation method^82,89^.

Our prepared vaccines were administered as one subcutaneous immunization followed by two intranasal boosts (s.c./i.n.). Combinatorial immunization, mainly systemic priming and mucosal-boosting can induce a humoral immune response in BALB/c mice^90–92^. High serum IgG and BAL IgA titers were recorded two weeks following the last booster dose compared to control groups receiving either PBS or CS:P NPs. Complement-mediated serum killing is an innate immune mechanism that protects the host from harmful bacteria. Serum sensitivity and resistance may be an important microbial characteristic that could potentially distinguish between invasive and non-invasive isolates^93^; *B.cenocepacia* K56-2 is a serum-resistance strain while *B.cenocepacia* J2315 is a serum-sensitive strains^93^. In our study, sera from vaccinated mice, collected two weeks following the last booster dose, neutralized *B. cenocepacia*. Similarly, neutralization of *B. cenocepacia* and cross-neutralization of *B. multivorans* with sera from mice vaccinated with mucosal OMP nanoemulsion was reported^94^.

Owing to the intrinsic ability of *B. cenocepacia* to survive and replicate within the host cells, both humoral and cell-mediated immune components are mandatory to combat Bcc pulmonary infections^94^. Thus, in addition to systemic and local antibody responses, we examined cell-mediated immunity triggered by proinflammatory cytokines, which generate protective immunity against Burkholderia infection^95^. TNF-α, a T helper (Th) 1 cytokine, is produced by various cells, including macrophages, T cells, and mast cells^96^, and plays a dual role in regulating protection and susceptibility to Burkholderia infection^97^. Th2 cells produce mainly IL-4, IL-5, IL-6, IL-10, and IL-13, which mediate B cell activation, antibody production, and the regulation of Th1 responses^98^. IL-6, an inflammatory cytokine produced by monocytes, macrophages, and endothelial cells, enhances innate immunity and antibody production post-mucosal vaccination^99^. Th17 cells act as a bridge between innate and adaptive immunity in the host’s defense against different pathogens at the mucosal surface. Activation of Th17 results in the production of IL-17A, IL-17F, and IL-22^100^. IL-17 has an important role in vaccine-induced immunity against respiratory infections, as it is vital for the clearance of respiratory pathogens^17,101^. Immunization with 6x-His-LY-CS:P NPs and 6x-His-KT-CS:P NPs activated Th1, Th2, and Th17 in antigen-restimulated splenocytes of vaccinated mice. Vaccination with *B. cenocepacia* Linocin or OmpW recombinant proteins was effective as stimulants of the cellular host response, with the production of IL-17A and IL-10^4^. Splenocytes from mice vaccinated with the live attenuated *B. pseudomallei* ΔtonB Δhcp1 (PBK001) strain showed increased production of IFN-γ, TNF-α, and IL-17A after stimulation with whole-cell lysate antigens^101^.

In our study, the immunized mice were challenged by intranasal administration of 2 × 10^8^ CFUs *B. cenocepacia* two weeks following the second booster dose. The results were promising, with all immunized mice showing a significant reduction in *B. cenocepacia* counts in the lungs compared to the PBS and CS:P NPs groups. Makidon and coworkers reported a decrease in *B. cenocepacia* counts in the lungs of mice immunized with an OMP nanoemulsion (OMP-NE) vaccine compared to control mice^94^. Similarly, intranasal vaccination with OMP and the adjuvant adamantylamide dipeptide induced mucosal immunity against *B*. *multivorans*, decreased lung colonization, and minimized tissue damage^16^. Overall, the analysis of immune stimulation response revealed that 6x-His-KT-CS:P NPs induced a higher level of IgA and IgG, as well as stronger cell-mediated immunity, compared to 6x-His-LY-CS:P NPs and 6x-His-BD-CS:P NPs, in that order.

In conclusion, reverse vaccinology proved efficient in saving the time and effort expended in identifying potential vaccine candidates for *B. cenocepacia*. Combinatorial systemic and intranasal immunization with 6x-His-LY-CS:P NPs, 6x-His-KT-CS:P NPs, or 6x-His-BD-CS:P NPs elicited a protective immune response against *B. cenocepacia* by inducing systemic, mucosal, and cellular immune responses. Furthermore, immunized mice were successfully protected against pulmonary colonization with *B. cenocepacia*. The 6x-His-KT-CS:P NPs induced high levels of mucosal antibodies, systemic antibodies, IL-17A, which may account for the improved clearance of *B. cenocepacia* from infected lungs. This provides new hope for saving the lives of those at high risk for developing *B. cenocepacia* infections. However, further studies are needed to validate its effectiveness against clinical *B. cenocepacia* isolates and other members of the Bcc and whether it could provide possible protection against infection in CF mice.

## Methods

### Bacterial strains and growth conditions

*B. cenocepacia* J2315 (DSM# 16553, Germany) was cultured using Lauria-Bertani (LB) broth and agar (Difco Laboratories, USA) at 30 °C for 48 h. The cloning host *Escherichia coli* (*E. coli*) TOP 10, and the expression host *E. coli* BL21 (DE3 strain) were obtained from (Invitrogen, CA, USA) and cultured in LB broth and agar at 37 °C for 18–24 h. When needed, the culture medium was supplemented with ampicillin (100 μg/mL) (EPICO Inc., Egypt). Bacterial strains were stored in LB broth containing 20% glycerol at −80 °C^16^.

### Bioinformatics analyses

The proteome of *B. cenocepacia* J2315 was retrieved from the Universal Protein (UniProt) database (Proteome ID: UP000001035) (http://www.uniprot.org/proteomes/)^72^ in FASTA format. The subcellular localization of the proteins was predicted using the PSORTb (v.3.0.2) online server (www.psort.org/psortb/)^102^. Extracellular and outer membrane proteins were screened using the Vaxign database (http://www.violinet.org/vaxign2)^103^ to exclude those exhibiting sequence homology to the host proteins (human and mouse) and predict their ability to adhere to the cells. The Expasy Protparam server was used to shortlist the selected proteins based on their physicochemical parameters: molecular weight and the instability index (https://web.expasy.org/protparam). Proteins were predicted as good vaccine targets when the cutoff value of their instability index was <40^104^.

The potential antigenicity of the shortlisted candidates was predicted using the VaxiJen 2.0 server (http://www.ddgpharmfac.net/vaxijen/VaxiJen/VaxiJen.html)^105^, with a threshold value ≥ 0.5. Prediction of the protein topology was performed using the transmembrane topology of protein helices determinant, TMHMM v2.0 server (http://www.cbs.dtu.dk/services/TMHMM/)^106^, and HMMTOP 2.0 (http://www.enzim.hu/hmmtop) with cutoff value ≤ 1 transmembrane domain^107^. Predicting the number of specific (IgG and IgA) B cell epitopes in the selected proteins was performed using the IgPred module (http://crdd.osdd.net/raghava/igpred/)^57^.

Shortlisted proteins containing IgG and IgA B cell epitopes were uploaded to the TepiTool (http://tools.iedb.org/tepitool) server of the Immune Epitope Database (IEDB)^108^ to perform T-cell epitopes prediction. We used the default parameters of IEDB to access the best binding 9-mer peptides in the protein sequences to a set of 27 of the most frequent A and B alleles (Supplementary Table 6) to predict MHC class I binders. The predicted peptide was chosen based on the predicted percentile rank with a cutoff value ≤ 1. We used the default parameters of IEDB, and the peptide length was fixed at 15-mers to detect the best binders to a panel of 26 of the most frequent alleles (Supplementary Table 6) to predict MHC class II binding epitopes. The predicted peptide was chosen based on the predicted percentile rank, with a cutoff value ≤ 10 as the default parameter^63^.

Conservation of candidate proteins in the most prevalent Bcc species: *B. cenocepacia, B. cepacia, B. multivorans, B. dolosa, B. stabilis, B. contaminans, and B. vietnamensis* was checked by the BLASTp search using the non-redundant protein sequence database from the National Center for Biotechnology Information (NCBI). The percentage identity and minimum query coverage were set to 80%; proteins showing sequence similarity <80% were excluded and considered non-conserved proteins. A schematic flowchart of the bioinformatics analysis is shown in Fig. 1.

Multiple sequence alignment of the three shortlisted protein candidates with representative strains of the most prevalent Bcc species was performed using Clustal Omega^109^ and visualized using the online program NCBI multiple sequence alignment viewer (version 1.25.1, NCBI, MD, USA). The “BLOSUM” method was used to show the level of similarity.

### Homology modeling

Homology models were built for the three shortlisted proteins using the SWISS MODEL (https://swissmodel.expasy.org/) online server^110^ and visualized by Chimera (https://www.cgl.ucsf.edu/chimera/)^111^ to highlight the epitope surface antigens for IgG and IgA in each protein.

### Cloning, expression, and purification of the selected protein candidates

Three protein candidates: alkaline phosphatase family protein WP_006481710.1 (LY), alkaline phosphatase family protein WP_012493605.1 (KT), and the hypothetical protein WP_006492970.1 (BD) were selected for further experiments. Genomic DNA was extracted from *B. cenocepacia* strain J2315 using the Wizard genomic DNA purification kit (Promega, USA). Oligonucleotide primers used in this study (Table 3) were designed using the NCBI Primer-BLAST tool and supplied by Macrogen, Korea. PCR amplification of the candidate genes BCAM1474, BCAM2678, and BCAM0384 using the respective primers was performed using ExTaq DNA polymerase (Takara, Japan). PCR products were purified using a QIAquick PCR purification kit (Qiagen, Germany).

**Table 3:** The sequences of the oligonucleotide primers used in the study and the expected amplicon size

Target gene	Primer sequence (5′-3′)	Amplicon size (bp)	Ta °C	Binding site	RE site
BCAM1474	LY001: GGAGTgaattcATGTTCCGTCAAGCCTTGCTCGTCA LY002: CGTCActcgagG ATCGACGGTGCGGCGCTCAATAC	1671	65	1638537–1640207	*EcoR*I *Xho*I
BCAM2678	KT001: GCA AgaattcATGGCGGCGATGGTGGCGGGTA KT002: AATTCCCCActcgagACGCCCGATACCGAGCCT	1644	62	3024228–3025871	*EcoR*I *Xho*I
BCAM0384	BD001: CGGTGaagcttATGAAAGCCGTTGCACTGATTGC BD002: ACGCGCCctcgagATTCTGCGTGAGCGGATTTA	1005	60.2	434497–435501	*Hind*III *Xho*I
pET22b -LY	LY003: CGAAGTAGCCCATCACCTGG LY004: TATGAAATACCTGCTGCCGACC	607	52	1327–1933	-
pET22b -KT	KT003: TCTTCACGTAGTTGTGCGGG KT004: TATGAAATACCTGCTGCCGACC	637	54	1270–1906	-
pET22b -BD	BD003: TCGTCGTGTACGTGTAGGTG DB004: TATGAAATACCTGCTGCCGACC	629	52	658–1286	-

The PCR purified products were subsequently double-digested with the respective restriction enzymes *EcoR*I, *Xho*I, and *Hind* III (Thermo Fisher Scientific, USA) and ligated into the expression vector pET22b (+) (Merck, Germany), using T4 DNA ligase (Takara, Japan). Electrocompetent *E. coli* TOP10 was transformed with the corresponding ligation reactions^112^. Transformants were selected on LB agar containing 100 μg/mL ampicillin and incubated at 37 °C for 24 h. Clones were confirmed with colony PCR using the primer pairs LY003-LY004, KT003-KT004, and BD003-BD004 for pET22b-LY, pET22b-KT, and pET22b-BD, respectively. Recombinant plasmids were extracted using the QIAprep Spin Miniprep kit (Qiagen, Germany) and transformed into electrocompetent *E. coli* BL21. Transformed colonies were selected on LB agar containing ampicillin (100 μg/mL) and incubated at 37 °C for 24 h.

An overnight culture of *E. coli* BL21 harboring the desired recombinant plasmid was sub-cultured into 50 mL Terrific broth (TB)^112^, containing (100 μg/mL) ampicillin, and incubated at 37 °C with shaking at 180 rpm to reach an optical density of 0.6–0.8 at 600 nm. Protein expression was induced by using 0.5 mM Isopropyl β-D-1-thiogalactopyranoside (IPTG) (Promega, USA) and incubated overnight at 30 °C with shaking at 180 rpm (pET system manual, Novagen, USA). Cells were harvested by centrifugation at 10,000 rpm for 10 min at 4 °C. Cell pellets were resuspended in native Lysis Buffer (50 mM NaH_2_PO_4_, 300 mM NaCl, 5 mM imidazole, pH 8.0) containing lysozyme (1 µg/mL), sonicated on ice for 30 min followed by centrifugation at 14,000 rpm for 20 min at 4 °C. 6x-His-tagged protein purification was carried out using Ni-NTA spin columns (Qiagen, Germany) under native conditions, according to the manufacturer’s instructions. The purity of the purified proteins was assessed using sodium dodecyl sulfate-polyacrylamide gel electrophoresis (SDS-PAGE). The purified fractions of each protein were pooled and dialyzed against 1× phosphate-buffered saline (PBS) (pH 7.2–7.4) using an electroeluter (BIO-RAD, USA)^113^. The protein concentration was estimated by the Bradford assay, using bovine serum albumin (BSA) (WINLAB Ltd., Leicestershire, UK) as standard^114^.

### Loading recombinant proteins onto chitosan-pectin nanoparticles

CS:P nanoparticles were prepared using the ionic gelation method^115,116^. Briefly, 1 mL of the recombinant protein (10 mg/mL) was added drop-wise to 1.5 mL (1 mg/mL) aqueous solution of pectin (El Nasr Co., Egypt) and stirred for 30 min at room temperature. Chitosan (low Mw, viscosity of 20 cps, and 85% deacetylation) (Sigma Aldrich, Germany) was dissolved in 1% v/v acetic acid to prepare a (2 mg/mL) solution, and the pH was adjusted to 5 using 1 N NaOH solution. The chitosan solution was added drop-wise to the prepared pectin-recombinant protein solution at a ratio of 2:1 with continuous stirring at 1000 rpm for 1 h at room temperature, and the resulting solution was subjected to low-speed centrifugation. CS:P-BSA was used as a non-specific antigen control. The nanoparticles were harvested by centrifugation at 14,000 rpm for 20 min at 4 °C, washed three times with MilliQ water, and freeze-dried (Freezone lyophilizer; Labconco Corporation, Kansas City, MO). The prepared CS:P nanoparticles were examined by a high-resolution transmission electron microscope JEM-2100 (JEOL, Japan). The particle size and zeta potential distribution of CS:P NPs were determined using the Malvern Zetasizer analyzer 2000 (Malvern Instruments Ltd., Malvern, UK).

The amount of free protein in the supernatant and wash was determined by Bradford protein assay^114^, and the amount of loaded antigen was calculated^117^.

### Immunization of mice using recombinant proteins loaded chitosan-pectin nanoparticles

#### Animals used in the study

Female BALB/c mice (*n* = 82, 6–8-week-old (20–25 g))^16^, were purchased from Theodor Bilharz Research Institute, Giza, Egypt, and were included in the study. Animals were housed under standard laboratory conditions at 20–25 °C, an average humidity of 50–60%, and a 12-h light/dark cycle. Food and water were ad libitum. The mice were allowed to acclimatize for one week. All procedures were performed according to the recommendations of the National Institutes of Health Guide for Care and Use of Laboratory Animals^118^ and were approved by the Ethics Committee of the Faculty of Pharmacy, Cairo University, Cairo, Egypt (Approval no: MI (2619)). Mice were weighed and monitored regularly throughout the experiment^119^.

### Validating *B. cenocepacia* lung colonization in BALB/c mice five days post-infection

Six mice were intranasally infected with approximately 2 × 10^8^ Colony-forming units (CFUs) of *B. cenocepacia* J2315 in 30 μL PBS, under thiopental anesthesia (EIPICO, Egypt), and six healthy mice received only PBS and served as control. Five days post-infection, mice were euthanized by an overdose of thiopental followed by cervical dislocation. The bacterial burden in the lungs was assessed in three mice from each group^16^, where the lungs were aseptically excised, homogenized, serially diluted, and plated. Plates were incubated at 37 °C for 72 h prior to manual CFU enumeration^120^. Lung tissue samples from the remaining infected and healthy control mice were dissected and fixed in 10% neutral buffered formalin for 72 h. Samples were processed in serial grades of ethanol, cleared in xylene, and then embedded in paraplast tissue embedding media. Full pulmonary tissue sections (4 μm thick) were cut by rotatory microtome and mounted on glass slides to demonstrate different lung lobes. At least three tissue sections per sample were stained by Harris Hematoxylin and Eosin as a general histological examination staining method and examined by an experienced histologist using a Full HD microscopic imaging system (Leica Microsystems GmbH, Germany)^121,122^.

### Immunization timeline in BALB/c mice

Mice were distributed into five groups, each comprising 14 mice. Each of the first three groups were subcutaneously immunized with 100 μg of one of the prepared recombinant proteins loaded CS:P NPs (6x-His-LY-CS:P NPs, 6x-His-KT-CS:P NPs, and 6x-His-BD-CS:P NPs) suspended in 50-μL PBS as a prime dose. The fourth group received 100 μg CS:P NPs suspended in 50-μL PBS, and the fifth group received 50-μL sterile endotoxin-free PBS. This was followed by two intranasal booster doses (50 μg/20-µL PBS), of the same used prime antigen, on days 14 and 28 (10 µl/nare) in anesthetized mice using thiopental (Fig. 6)^123^.

### Determination of serum IgG and IgA in bronchoalveolar lavage

Seven mice from each group were anesthetized two weeks following the second booster dose (day 42) (Fig. 6). The BAL fluid was collected and centrifuged at 3000 rpm for 5 min to remove cellular debris^16^. Blood samples were collected by cardiac puncture and allowed to clot at room temperature; the sera were collected by centrifugation at 3000 rpm for 15 min and stored at −20 °C^4^. Serum IgG and BAL IgA levels were determined by indirect enzyme-linked immunosorbent assay (ELISA)^124^. Flat-bottom ELISA 96-well microplates (Greiner Bio-One) were coated with 100 µL of the purified protein solutions in sodium carbonate buffer (pH 9.5) at concentrations of 15 µg/mL and 20 µg/mL for determination of IgG and IgA, respectively. After washing with PBS containing 0.1% Tween 20 (PBST) and blocking with 5% skimmed milk in PBST, two-fold serially diluted sera or BAL were added, and the plates were incubated at 37 °C for 2 h in case of sera and overnight at 4 °C for BAL. Following washing, horseradish peroxidase (HRP) conjugated goat anti-mouse IgG (Thermo Fisher Scientific, USA), (1:2000 dilution) or HRP- conjugated goat anti-mouse IgA antibody (Thermo Fisher Scientific, USA) (1:2000 dilution) were added. The absorbance of the color produced after the addition of 3,3′,5,5′-tetramethylbenzidine (TMB) (Thermo Fisher Scientific, USA) was measured at 450 nm, using an ELISA plate reader (BioTek Instruments). Antibody titers were defined as the reciprocal of the highest dilution of serum or BAL producing an absorbance above the cutoff value, where the cutoff value was the absorbance of the corresponding dilution of serum or BAL of unvaccinated mice (PBS group) +3 standard deviations.

### Splenocyte culture and analysis of cytokines expression

Spleens were aseptically excised and processed from three mice from each immunized and control group two weeks following the second booster dose (day 42) (Fig. 6)^94,125^. Briefly, the spleen tissue was sliced into 2 mm slices and transferred to a sterile 24-well plate with a flat bottom containing 1 mL Dulbecco’s modified Eagle medium (DMEM) (Gibco, Thermo Fisher Scientific, Germany) containing 10% fetal bovine serum (FBS) (Gibco, Thermo Fisher Scientific, Germany), 1% of penicillin G sodium (10.000 UI), streptomycin (10 mg) and amphotericin B (25 μg) (PSA) (Gibco, Thermo Fisher Scientific, Germany). Culture plates were incubated at 37 °C in an atmosphere of 5% CO_2_ for 24 h. The next day, 50 µg/mL of the recombinant protein-loaded CS:P NPs, CS:P NPs, and PBS buffer was added to the corresponding cultured spleen cells. The treated cells were incubated at 37 °C in an atmosphere of 5% CO_2_ for 72 h. At the end of incubation, the conditioned media was collected from each well, and the concentration of IL-17A, IL-6, and TNF-α was measured using ELISA kits, according to the manufacturer’s instructions (Elabscience Biotechnology, USA).

### Testing serum antibody bactericidal activity in vaccinated mice

Assessment of the bactericidal activity of serum antibodies in vaccinated mice was determined according to Makidon and coworkers^94^. Briefly, 50 μL of serially diluted pooled sera from each vaccinated mice group, the adjuvant group, and naïve mice; were each incubated with 50 μL *B. cenocepacia* J2315 cultures (3 × 10^4^ CFU) in Mueller-Hinton broth (MHB) (Difco Laboratories) in a 96-well microtiter plates at 37 °C. The viability of *B. cenocepacia* J2315 was determined by viable counting after 48 h; the experiment was performed in triplicate^120^. Wells containing 100 μL MHB inoculated with 3 × 10^4^ CFU *B. cenocepacia* J2315 served as a growth control.

### Pulmonary clearance challenge

The bacterial challenge studies were performed on the remaining mice from all groups (*n* = 7/group)^126^. Briefly, three weeks after the last intranasal booster dose, mice were anesthetized and infected intranasally with approximately 2 × 10^8^ CFU *B. cenocepacia* J2315 in 30 μL PBS. Five days post-infection, mice were euthanized by an overdose of thiopental, followed by cervical dislocation. The lungs were aseptically excised, homogenized, serially diluted, and plated. Plates were incubated at 37 °C for 72 h prior to manual CFU enumeration^120^.

### Statistical analysis

Statistical analyses were performed using Graph Pad Prism v9 software. A one-way analysis of variance (ANOVA) test followed by Dunnett’s multiple comparisons test was used to analyze the results of cytokines expression, bactericidal activity of serum antibodies, and pulmonary clearance experiments. An unpaired t-test was used to determine the significant differences in antibody (IgG and IgA) titers. The data obtained were represented as mean ± SD. Results with a *p-*value < 0.05 were considered significant.

## Supplementary information

Supplementary information

## Data Availability

All data supporting the findings of this study are included in the main manuscript or the supplementary material. Other data included in this study will be available upon request.
