# Supplementary material for: Cyclic di AMP phosphodiesterase nanovaccine elicits protective immunity against Burkholderia cenocepacia infection in mice
Source: NPJ Vaccines. 2025 Feb 1;10:22. doi: 10.1038/s41541-025-01074-4 (PMC11787396; doi:10.1038/s41541-025-01074-4)
Supplement: Supplementary file 1 — Supplementary information [file 41541_2025_1074_MOESM1_ESM.pdf]

NCBI Multiple Sequence Alignment Viewer, Version 1.25.0

LY-MSA

Alignment

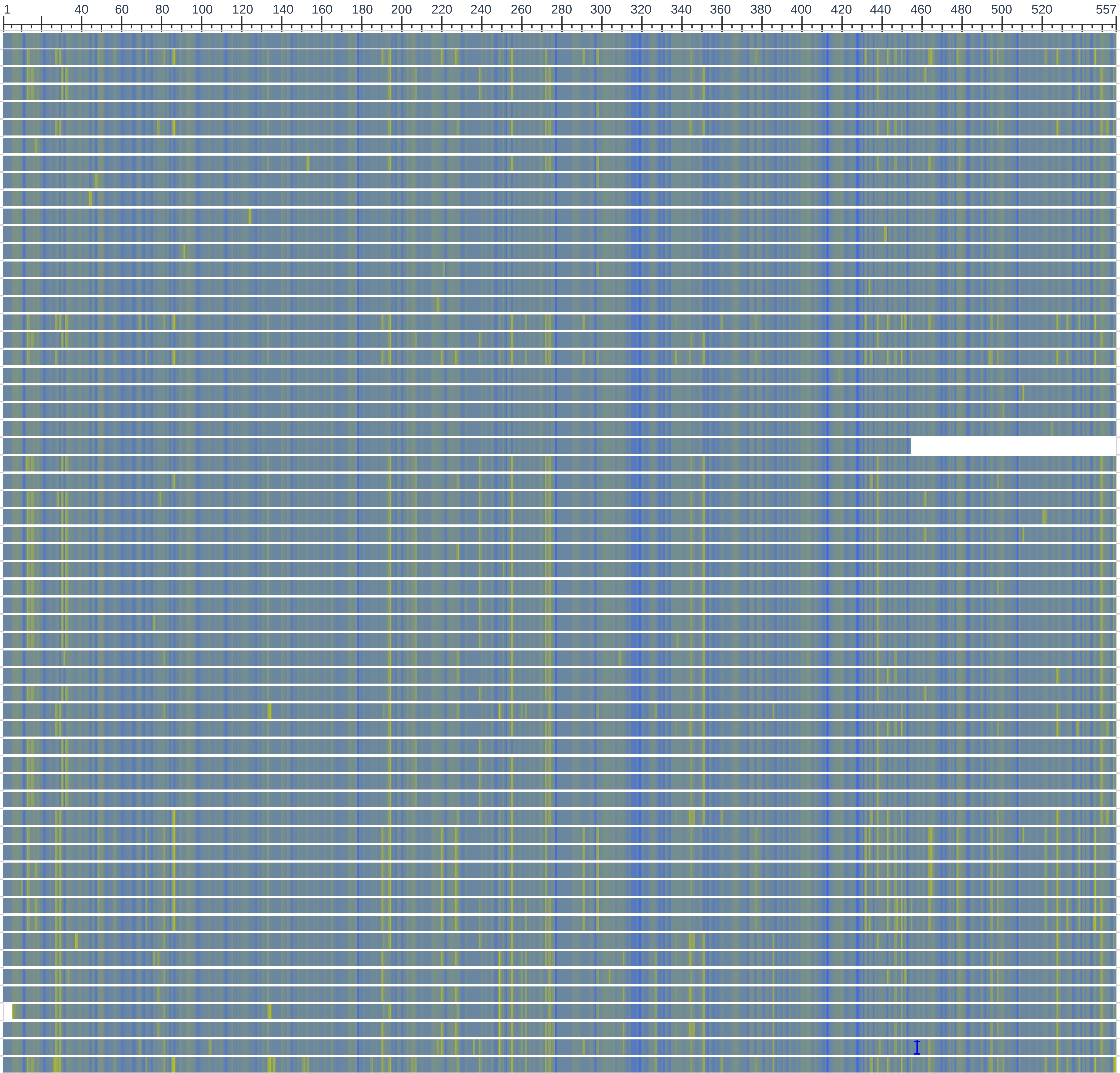

Sequence ID	Start	End	Organism
WP_006481710.1 (+)	1	557	Burkholderia
WP_006765888.1 (+)	1	557	Burkholderia
WP_011547745.1 (+)	1	557	Burkholderia
WP_175878585.1 (+)	1	557	Burkholderia
WP_058903100.1 (+)	1	557	Burkholderia
WP_039367329.1 (+)	1	557	Burkholderia
WP_006494895.1 (+)	1	557	Burkholderia cenocepacia
HEF5869782.1 (+)	1	557	Burkholderia cenocepacia
WP_321947217.1 (+)	1	557	Burkholderia cenocepacia
WP_006491362.1 (+)	1	557	Burkholderia cenocepacia
WP_200079000.1 (+)	1	557	Burkholderia cenocepacia
WP_080331871.1 (+)	1	557	Burkholderia cenocepacia
WP_069352237.1 (+)	1	557	Burkholderia cenocepacia
WP_272543246.1 (+)	1	557	Burkholderia cenocepacia
WP_212202935.1 (+)	1	557	Burkholderia cenocepacia
WP_124550687.1 (+)	1	557	Burkholderia cenocepacia
AIO39339.1 (+)	1	557	Burkholderia cenocepacia
WP_326850809.1 (+)	1	557	Burkholderia cenocepacia
AIO34865.1 (+)	1	557	Burkholderia cenocepacia
WP_212261336.1 (+)	1	557	Burkholderia cenocepacia
WP_212247168.1 (+)	1	557	Burkholderia cenocepacia
WP_200062050.1 (+)	1	557	Burkholderia cenocepacia
WP_241338803.1 (+)	1	557	Burkholderia cenocepacia
WP_049124175.1 (+)	1	454	Burkholderia cenocepacia
WP_175767447.1 (+)	1	557	Burkholderia cenocepacia
WP_050011020.1 (+)	1	557	Burkholderia cenocepacia
HEM7882926.1 (+)	1	557	Burkholderia cenocepacia
WP_323612294.1 (+)	1	557	Burkholderia cenocepacia
WP_310643666.1 (+)	1	557	Burkholderia cenocepacia
WP_077219214.1 (+)	1	557	Burkholderia cenocepacia
WP_212146594.1 (+)	1	557	Burkholderia cenocepacia
WP_212041322.1 (+)	1	557	Burkholderia cenocepacia
WP_175833771.1 (+)	1	557	Burkholderia cenocepacia
WP_124461051.1 (+)	1	557	Burkholderia cenocepacia
WP_202754421.1 (+)	1	557	Burkholderia cenocepacia
WP_321856757.1 (+)	1	557	Burkholderia cenocepacia
WP_126369924.1 (+)	1	557	Burkholderia cenocepacia
EAY64939.1 (+)	31	587	Burkholderia cenocepac...
WP_047903331.1 (+)	1	557	Burkholderia cepacia c...
WP_175238707.1 (+)	1	557	Burkholderia cepacia c...
WP_212181553.1 (+)	1	557	Burkholderia cepacia c...
WP_012340068.1 (+)	1	557	Burkholderia cepacia c...
WP_212239134.1 (+)	1	557	Burkholderia cepacia c...
WP_023475717.1 (+)	1	557	Burkholderia cepacia c...
WP_046547756.1 (+)	1	557	Burkholderia contaminans
WP_176075194.1 (+)	1	557	Burkholderia dolosa
WP_212154135.1 (+)	1	557	Burkholderia dolosa
WP_212177757.1 (+)	1	557	Burkholderia dolosa
WP_212220654.1 (+)	1	557	Burkholderia dolosa
WP_006403585.1 (+)	1	556	Burkholderia multivorans
WP_006415229.1 (+)	1	556	Burkholderia multivorans
WP_021159176.1 (+)	1	557	Burkholderia sp. AU4i
WP_122169539.1 (+)	1	557	Burkholderia stabilis
WP_096474427.1 (+)	1	557	Burkholderia stabilis
WP_069748153.1 (+)	1	557	Burkholderia stabilis
GAU04437.1 (+)	1	552	Burkholderia stabilis
WP_176039464.1 (+)	1	557	Burkholderia stabilis
WP_129514844.1 (+)	1	559	Burkholderia stabilis
ABO57835.1 (+)	1	557	Burkholderia vietnamie...

NCBI Multiple Sequence Alignment Viewer, Version 1.25.0

KT-MSA

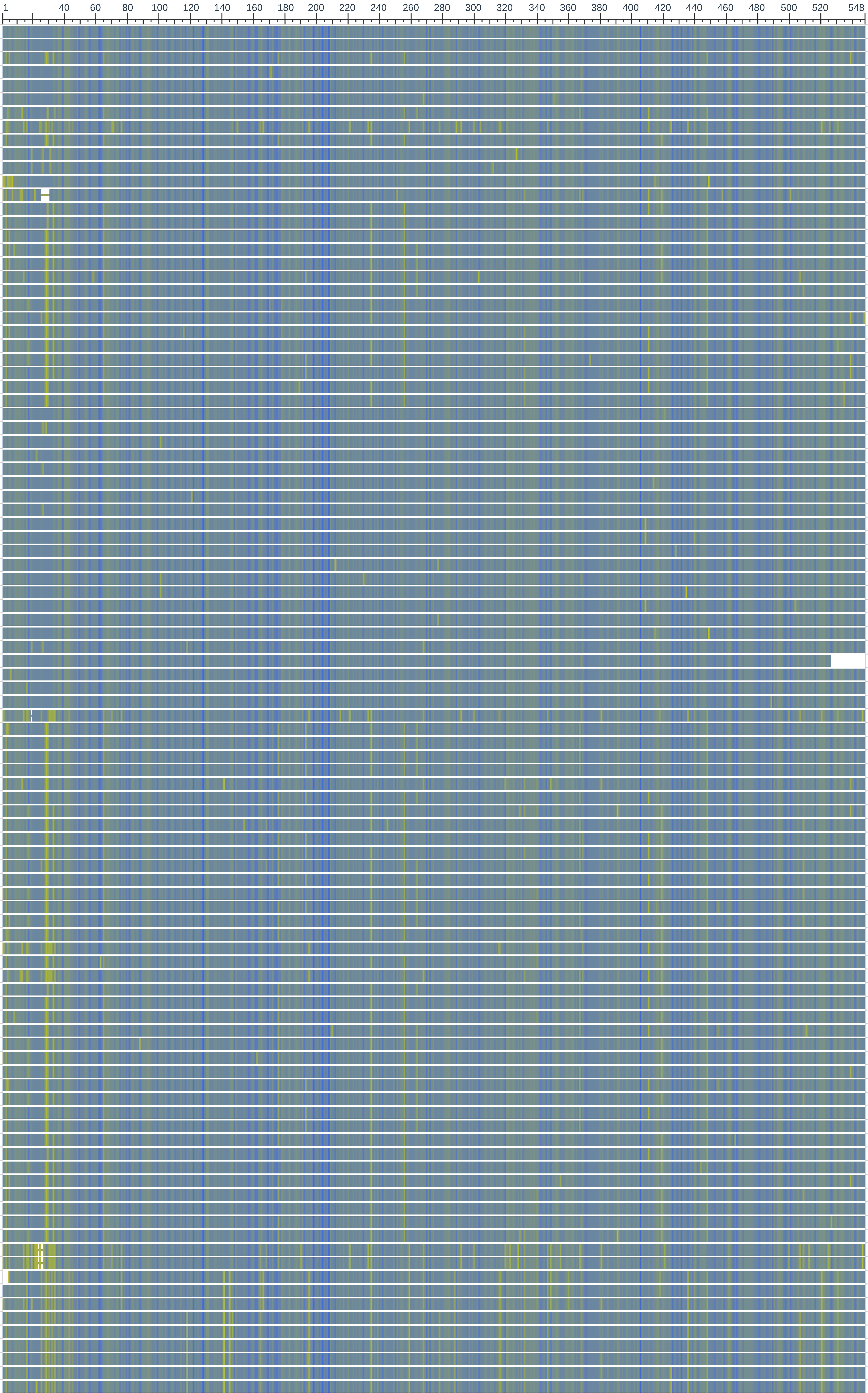

Sequence ID	Start	End	Organism
CAR56542.1 (+)	1	548	Burkholderia cenocepac...
WP_006487965.1 (+)	11	558	Burkholderia
WP_011694882.1 (+)	11	558	Burkholderia
WP_059722151.1 (+)	11	558	Burkholderia
WP_105865869.1 (+)	11	558	Burkholderia
WP_077188716.1 (+)	11	558	Burkholderia
WP_175878304.1 (+)	11	558	Burkholderia
WP_129515967.1 (+)	11	558	Burkholderia
HEB3533227.1 (+)	11	558	Burkholderia cenocepacia
WP_212261016.1 (+)	11	558	Burkholderia cenocepacia
WP_109342902.1 (+)	11	558	Burkholderia cenocepacia
WP_077234476.1 (+)	4	551	Burkholderia cenocepacia
WP_050012441.1 (+)	11	552	Burkholderia cenocepacia
WP_200094077.1 (+)	11	558	Burkholderia cenocepacia
AOJ19947.1 (+)	11	558	Burkholderia cenocepacia
WP_212171993.1 (+)	11	558	Burkholderia cenocepacia
WP_212044738.1 (+)	11	558	Burkholderia cenocepacia
WP_175681727.1 (+)	11	558	Burkholderia cenocepacia
WP_212176986.1 (+)	11	558	Burkholderia cenocepacia
HDR9800854.1 (+)	11	558	Burkholderia cenocepacia
WP_077220705.1 (+)	11	558	Burkholderia cenocepacia
WP_323615181.1 (+)	11	558	Burkholderia cenocepacia
WP_175768305.1 (+)	11	558	Burkholderia cenocepacia
WP_212194030.1 (+)	11	558	Burkholderia cenocepacia
WP_124548659.1 (+)	11	558	Burkholderia cenocepacia
WP_124698887.1 (+)	11	558	Burkholderia cenocepacia
WP_226110435.1 (+)	11	558	Burkholderia cenocepacia
WP_202756117.1 (+)	11	558	Burkholderia cenocepacia
WP_212191114.1 (+)	11	558	Burkholderia cenocepacia
WP_310640524.1 (+)	11	558	Burkholderia cenocepacia
WP_321950142.1 (+)	11	558	Burkholderia cenocepacia
WP_310628913.1 (+)	11	558	Burkholderia cenocepacia
WP_301828419.1 (+)	11	558	Burkholderia cenocepacia
WP_212250085.1 (+)	11	558	Burkholderia cenocepacia
WP_212158292.1 (+)	11	558	Burkholderia cenocepacia
WP_321853604.1 (+)	11	558	Burkholderia cenocepacia
WP_265224489.1 (+)	11	558	Burkholderia cenocepacia
WP_062910191.1 (+)	11	558	Burkholderia cenocepacia
WP_205671645.1 (+)	11	558	Burkholderia cenocepacia
WP_006494371.1 (+)	11	558	Burkholderia cenocepacia
WP_212203507.1 (+)	11	558	Burkholderia cenocepacia
WP_200037849.1 (+)	11	558	Burkholderia cenocepacia
WP_112750383.1 (+)	11	558	Burkholderia cenocepacia
WP_301794036.1 (+)	11	558	Burkholderia cenocepacia
WP_058903547.1 (+)	11	558	Burkholderia cenocepacia
WP_212094009.1 (+)	11	558	Burkholderia cenocepacia
ODN63287.1 (+)	11	536	Burkholderia cenocepacia
WP_323631802.1 (+)	11	558	Burkholderia cenocepacia
WP_077021394.1 (+)	11	558	Burkholderia cenocepacia
WP_322075317.1 (+)	11	558	Burkholderia cenocepacia
AIO36333.1 (+)	11	557	Burkholderia cenocepacia
WP_234621538.1 (+)	11	558	Burkholderia cenocepacia
WP_310643083.1 (+)	11	558	Burkholderia cenocepacia
ELK7720313.1 (+)	11	558	Burkholderia cenocepacia
WP_034175097.1 (+)	11	558	Burkholderia cenocepacia
HEF5875711.1 (+)	11	558	Burkholderia cenocepacia
WP_043888631.1 (+)	11	558	Burkholderia cenocepacia
WP_124677309.1 (+)	11	558	Burkholderia cenocepacia
WP_053525064.1 (+)	11	558	Burkholderia cenocepacia
WP_175787326.1 (+)	11	558	Burkholderia cenocepacia
WP_175777794.1 (+)	11	558	Burkholderia cenocepacia
HDR9807721.1 (+)	11	558	Burkholderia cenocepacia
WP_310640620.1 (+)	11	558	Burkholderia cenocepacia
WP_074805135.1 (+)	11	558	Burkholderia cenocepacia
WP_124476293.1 (+)	11	558	Burkholderia cenocepacia
WP_226133242.1 (+)	11	558	Burkholderia cenocepacia
WP_226248334.1 (+)	11	558	Burkholderia cenocepacia
WP_321859981.1 (+)	11	558	Burkholderia cenocepacia
WP_212211331.1 (+)	11	558	Burkholderia cenocepacia
WP_126368501.1 (+)	11	558	Burkholderia cenocepacia
WP_241331349.1 (+)	11	558	Burkholderia cenocepacia
WP_321957698.1 (+)	11	558	Burkholderia cenocepacia
WP_226142724.1 (+)	11	558	Burkholderia cenocepacia
WP_226240745.1 (+)	11	558	Burkholderia cenocepacia
WP_310623713.1 (+)	11	558	Burkholderia cenocepacia
WP_027809407.1 (+)	11	558	Burkholderia cenocepacia
WP_212145614.1 (+)	11	558	Burkholderia cenocepacia
WP_326860603.1 (+)	11	558	Burkholderia cenocepacia
WP_175831816.1 (+)	11	558	Burkholderia cenocepacia
ESS36799.1 (+)	1	548	Burkholderia cenocepac...
EAY66182.1 (+)	12	559	Burkholderia cenocepac...
WP_077179857.1 (+)	11	558	Burkholderia cepacia c...
WP_212103967.1 (+)	11	558	Burkholderia cepacia c...
WP_212108122.1 (+)	11	558	Burkholderia cepacia c...
WP_011548934.1 (+)	11	558	Burkholderia cepacia c...
WP_212181997.1 (+)	11	558	Burkholderia cepacia c...
WP_012339256.1 (+)	11	558	Burkholderia cepacia c...
WP_212050064.1 (+)	11	558	Burkholderia cepacia c...
WP_124466561.1 (+)	11	558	Burkholderia cepacia c...
WP_043280430.1 (+)	11	555	Burkholderia multivorans
EJO62038.1 (+)	12	556	Burkholderia multivora...
GAU06065.1 (+)	1	544	Burkholderia stabilis
WP_065503447.1 (+)	11	558	Burkholderia stabilis
WP_096473302.1 (+)	11	558	Burkholderia stabilis
WP_069749319.1 (+)	11	557	Burkholderia stabilis
WP_175995747.1 (+)	11	557	Burkholderia stabilis
WP_176041441.1 (+)	11	557	Burkholderia stabilis
HDR9584752.1 (+)	11	557	Burkholderia stabilis
WP_241302122.1 (+)	11	557	Burkholderia stabilis
WP_122170520.1 (+)	11	558	Burkholderia stabilis

NCBI Multiple Sequence Alignment Viewer, Version 1.25.0

BD-MSA

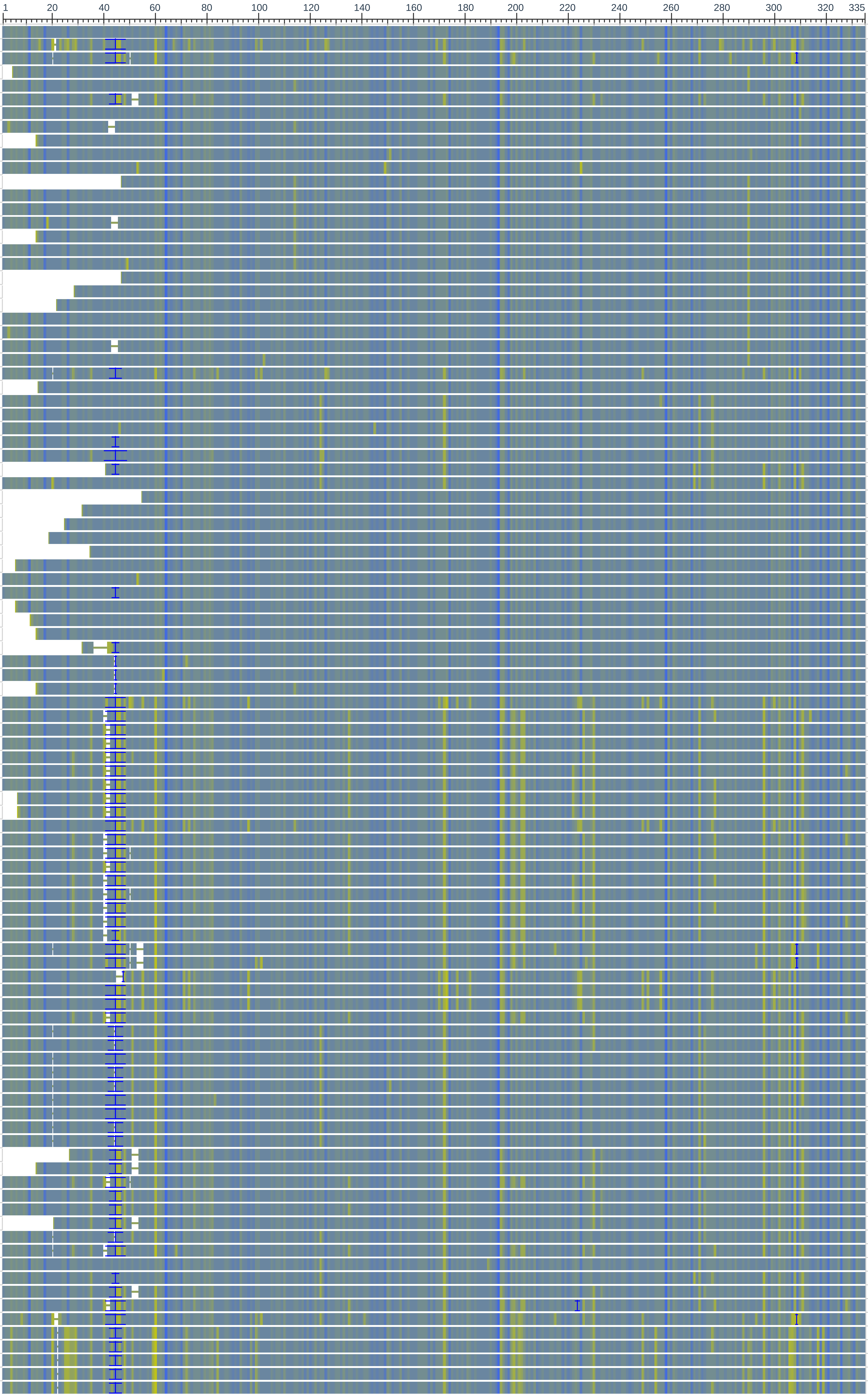

Sequence ID	Start	Alignment	End	Organism
WP_006492970.1 (+)	1		335	Burkholderia
WP_039369763.1 (+)	1		342	Burkholderia
WP_175876729.1 (+)	1		342	Burkholderia
WP_163123233.1 (+)	1		331	Burkholderia
WP_111441796.1 (+)	1		335	Burkholderia
WP_011548425.1 (+)	1		337	Burkholderia
WP_077021464.1 (+)	1		335	Burkholderia cenocepacia
WP_301834025.1 (+)	1		332	Burkholderia cenocepacia
WP_251233943.1 (+)	1		322	Burkholderia cenocepacia
WP_310628842.1 (+)	1		335	Burkholderia cenocepacia
WP_275525176.1 (+)	1		335	Burkholderia cenocepacia
WP_321903762.1 (+)	1		289	Burkholderia cenocepacia
CAB5126587.1 (+)	21		355	Burkholderia cenocepacia
RAS56873.1 (+)	21		355	Burkholderia cenocepacia
WP_317161795.1 (+)	1		332	Burkholderia cenocepacia
WP_321884675.1 (+)	1		322	Burkholderia cenocepacia
WP_212093860.1 (+)	1		335	Burkholderia cenocepacia
WP_212126844.1 (+)	1		335	Burkholderia cenocepacia
WP_321853845.1 (+)	1		289	Burkholderia cenocepacia
WP_241313406.1 (+)	1		307	Burkholderia cenocepacia
WP_059722252.1 (+)	1		314	Burkholderia cenocepacia
WP_109342028.1 (+)	1		335	Burkholderia cenocepacia
WP_249486156.1 (+)	1		335	Burkholderia cenocepacia
WP_321906346.1 (+)	1		332	Burkholderia cenocepacia
WP_212214515.1 (+)	1		335	Burkholderia cenocepacia
WP_050018242.1 (+)	1		339	Burkholderia cenocepacia
HEM7806447.1 (+)	1		321	Burkholderia cenocepacia
WP_175778310.1 (+)	1		335	Burkholderia cenocepacia
WP_124464779.1 (+)	1		335	Burkholderia cenocepacia
WP_212193845.1 (+)	1		335	Burkholderia cenocepacia
WP_175769352.1 (+)	1		338	Burkholderia cenocepacia
WP_175805991.1 (+)	1		344	Burkholderia cenocepacia
HEM7884268.1 (+)	1		298	Burkholderia cenocepacia
WP_212107865.1 (+)	1		335	Burkholderia cenocepacia
WP_077238679.1 (+)	1		281	Burkholderia cenocepacia
WP_270957230.1 (+)	1		304	Burkholderia cenocepacia
WP_164530858.1 (+)	1		311	Burkholderia cenocepacia
ELW9532708.1 (+)	1		317	Burkholderia cenocepacia
WP_077199502.1 (+)	1		301	Burkholderia cenocepacia
WP_260433962.1 (+)	1		330	Burkholderia cenocepacia
WP_060263099.1 (+)	1		335	Burkholderia cenocepacia
WP_112750953.1 (+)	1		338	Burkholderia cenocepacia
MCW3588153.1 (+)	1		330	Burkholderia cenocepacia
WP_321929207.1 (+)	1		324	Burkholderia cenocepacia
WP_241301350.1 (+)	1		322	Burkholderia cenocepacia
WP_321892791.1 (+)	1		301	Burkholderia cenocepacia
WP_212167874.1 (+)	1		335	Burkholderia cenocepacia
WP_034203413.1 (+)	1		335	Burkholderia cenocepacia
WP_321875921.1 (+)	1		322	Burkholderia cenocepacia
WP_301830383.1 (+)	1		343	Burkholderia cenocepacia
WP_212144924.1 (+)	1		341	Burkholderia cenocepacia
WP_205673747.1 (+)	1		341	Burkholderia cenocepacia
WP_077219859.1 (+)	1		341	Burkholderia cenocepacia
WP_226139645.1 (+)	1		341	Burkholderia cenocepacia
WP_175681927.1 (+)	1		341	Burkholderia cenocepacia
WP_212042441.1 (+)	1		341	Burkholderia cenocepacia
WP_154585942.1 (+)	1		335	Burkholderia cenocepacia
KOR17288.1 (+)	1		335	Burkholderia cenocepacia
WP_212249738.1 (+)	1		343	Burkholderia cenocepacia
WP_226132958.1 (+)	1		341	Burkholderia cenocepacia
AOJ20108.1 (+)	1		340	Burkholderia cenocepacia
WP_212169452.1 (+)	1		341	Burkholderia cenocepacia
HDR9804690.1 (+)	1		341	Burkholderia cenocepacia
WP_212176178.1 (+)	1		340	Burkholderia cenocepacia
WP_027807694.1 (+)	1		341	Burkholderia cenocepacia
WP_212204003.1 (+)	1		341	Burkholderia cenocepacia
WP_175786948.1 (+)	1		336	Burkholderia cenocepacia
WP_124702029.1 (+)	1		339	Burkholderia cenocepacia
WP_226105266.1 (+)	1		340	Burkholderia cenocepacia
MDI9650812.1 (+)	1		333	Burkholderia cenocepacia
WP_046338130.1 (+)	1		343	Burkholderia cenocepacia
WP_077189287.1 (+)	1		343	Burkholderia cenocepacia
WP_175832169.1 (+)	1		341	Burkholderia cenocepacia
WP_212158166.1 (+)	1		339	Burkholderia cenocepacia
WP_323661626.1 (+)	1		340	Burkholderia cenocepacia
WP_124478455.1 (+)	1		342	Burkholderia cenocepacia
WP_265225730.1 (+)	1		339	Burkholderia cenocepacia
WP_326862819.1 (+)	1		339	Burkholderia cenocepacia
WP_226239529.1 (+)	1		342	Burkholderia cenocepacia
WP_310641966.1 (+)	1		342	Burkholderia cenocepacia
WP_043888005.1 (+)	1		339	Burkholderia cenocepacia
WP_310644211.1 (+)	1		339	Burkholderia cenocepacia
WP_200075337.1 (+)	1		311	Burkholderia cenocepacia
HEM7903107.1 (+)	1		324	Burkholderia cenocepacia
WP_074804536.1 (+)	1		340	Burkholderia cenocepacia
WP_321958931.1 (+)	1		340	Burkholderia cenocepacia
WP_311012061.1 (+)	1		340	Burkholderia cenocepacia
WP_322090240.1 (+)	1		317	Burkholderia cenocepacia
ESS38239.1 (+)	28		366	Burkholderia cenocepac...
WP_212050407.1 (+)	1		340	Burkholderia cepacia c...
WP_124484577.1 (+)	1		335	Burkholderia cepacia c...
WP_105797970.1 (+)	1		338	Burkholderia cepacia c...
WP_102961941.1 (+)	1		337	Burkholderia cepacia c...
WP_212183092.1 (+)	1		343	Burkholderia cepacia c...
WP_046549083.1 (+)	1		342	Burkholderia contaminans
WP_241294150.1 (+)	1		339	Burkholderia stabilis
WP_241300141.1 (+)	1		339	Burkholderia stabilis
WP_175995500.1 (+)	1		339	Burkholderia stabilis
HDR9587977.1 (+)	1		339	Burkholderia stabilis
WP_069750421.1 (+)	1		339	Burkholderia stabilis

Supplementary figure 1: Visualization of multiple alignments for LY (WP_006481710.1), KT (WP_012493605.1), and BD (WP_006492970.1) proteins with representative strains of the most prevalent Bcc species (top 20 sequences) using the online program NCBI Multiple Sequence Alignment Viewer (version 1.20.1, NCBI, MD, USA). The coloring in each column reflects the match score to the residue on the consensus sequence: blue indicates a higher degree of matching, while green indicates a lower degree of matching.

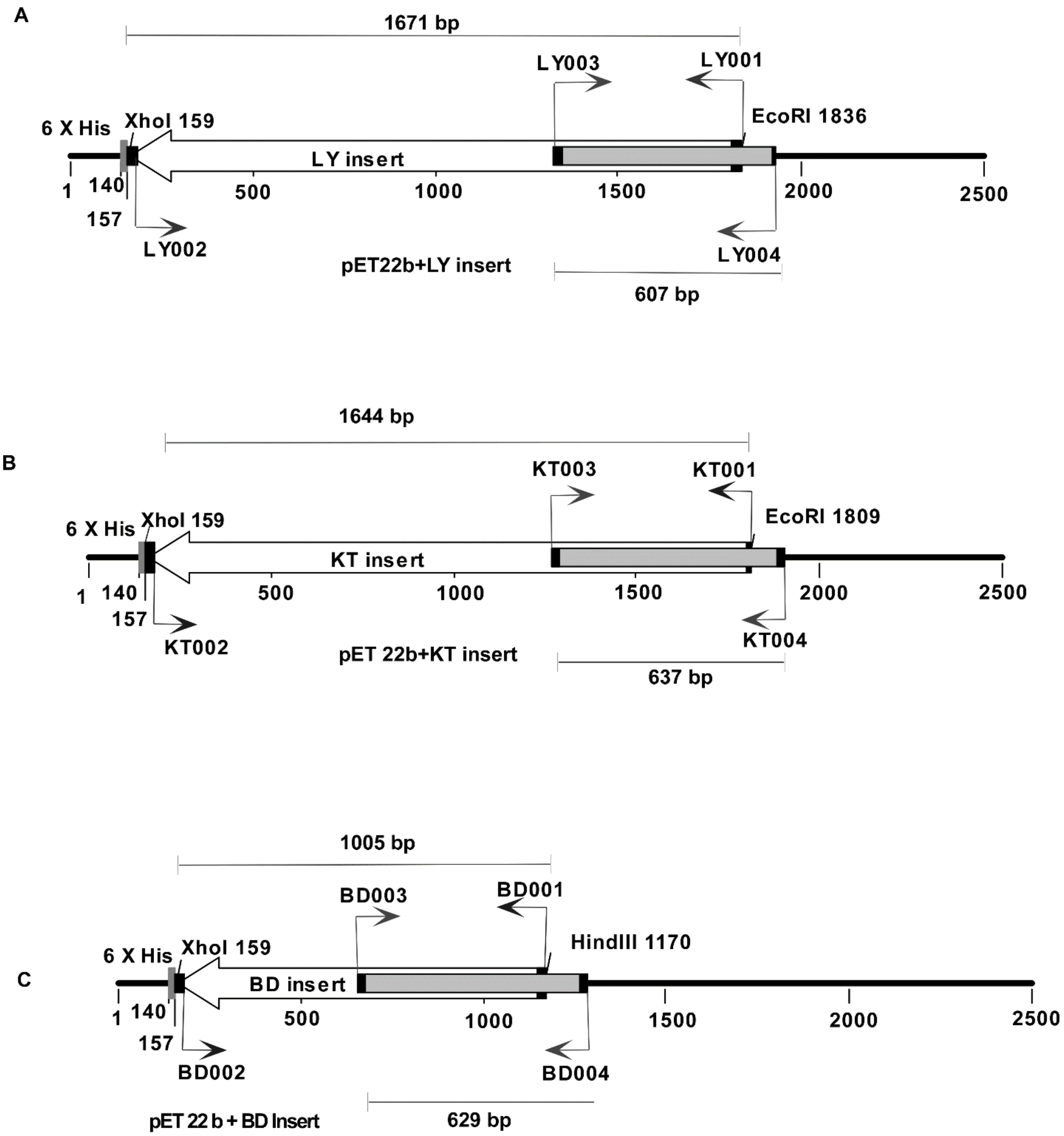

Supplementary figure 2. **Schematic representation of the cloning site of the expression vector pET22b+ harboring the genes of interest.**

Schematic diagram generated by BioEdit (7.2.5., 2013) of pET22b vector containing the different inserts with the position of different primers highlighted where: (A) pET22b + vector containing LY insert, (B) pET22b + vector containing KT insert, (C) pET22b + vector containing BD.

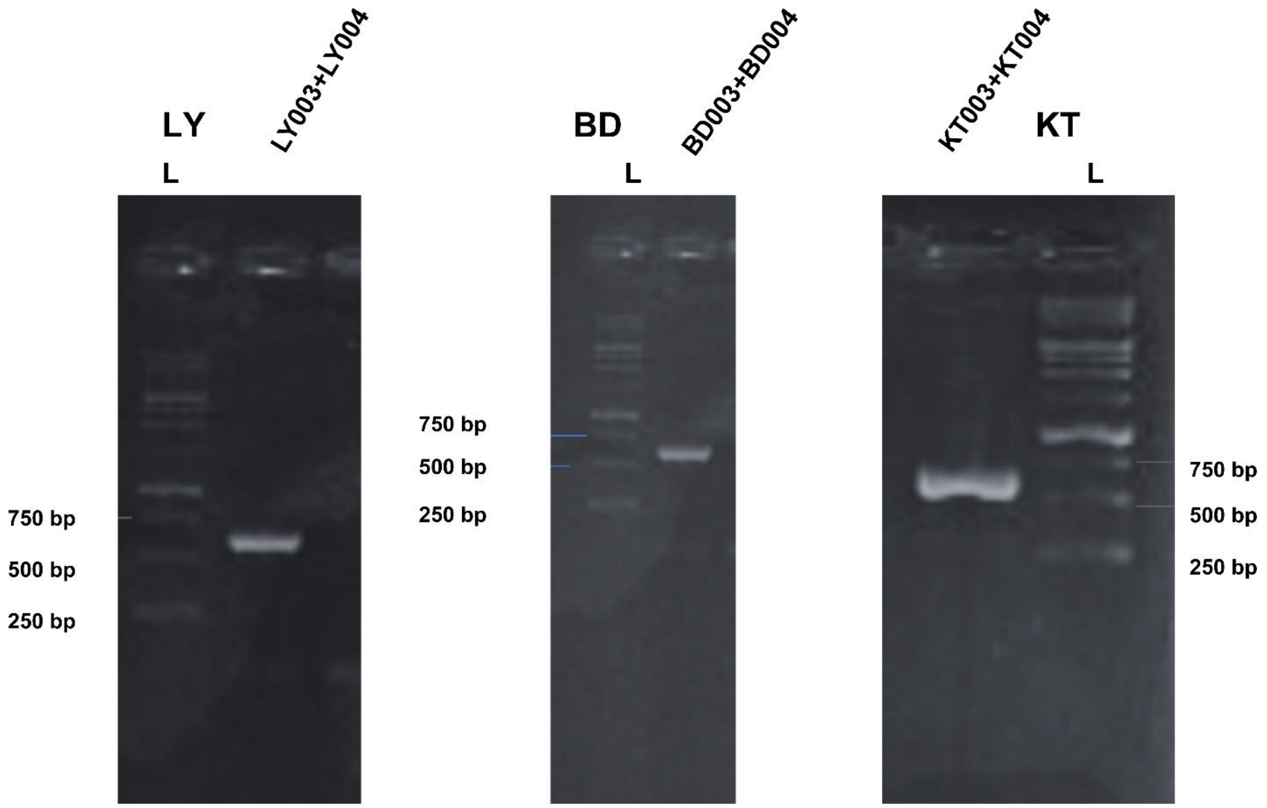

Supplementary Figure 3. **Confirmation of successful cloning.**

A photograph of 0.8% (wt/vol) agarose gel showing recombinant clone analysis of the PCR amplification products for screening for the correct clone using the following primers: (LY003-LY004), (KT003-KT004), and (BD003-BD004) where Lane L: GeneDireX 1 kb DNA Ladder (250 bp, 500 bp, 750 bp, 1000 bp, 1500 bp, 2000 bp, 2500 bp, 3000 bp, 3500 bp, 4000 bp, 5000 bp, 6000 bp, 8000 bp, 10000 bp), Lane (LY003-LY004): correct orientation of LY insert with the expected product size of 607 bp. Lane (BD003-BD004): correct orientation of BD insert with the expected product size of 629 bp, Lane (KT003-KT004): correct orientation of KT insert with the expected product size of 637 bp.

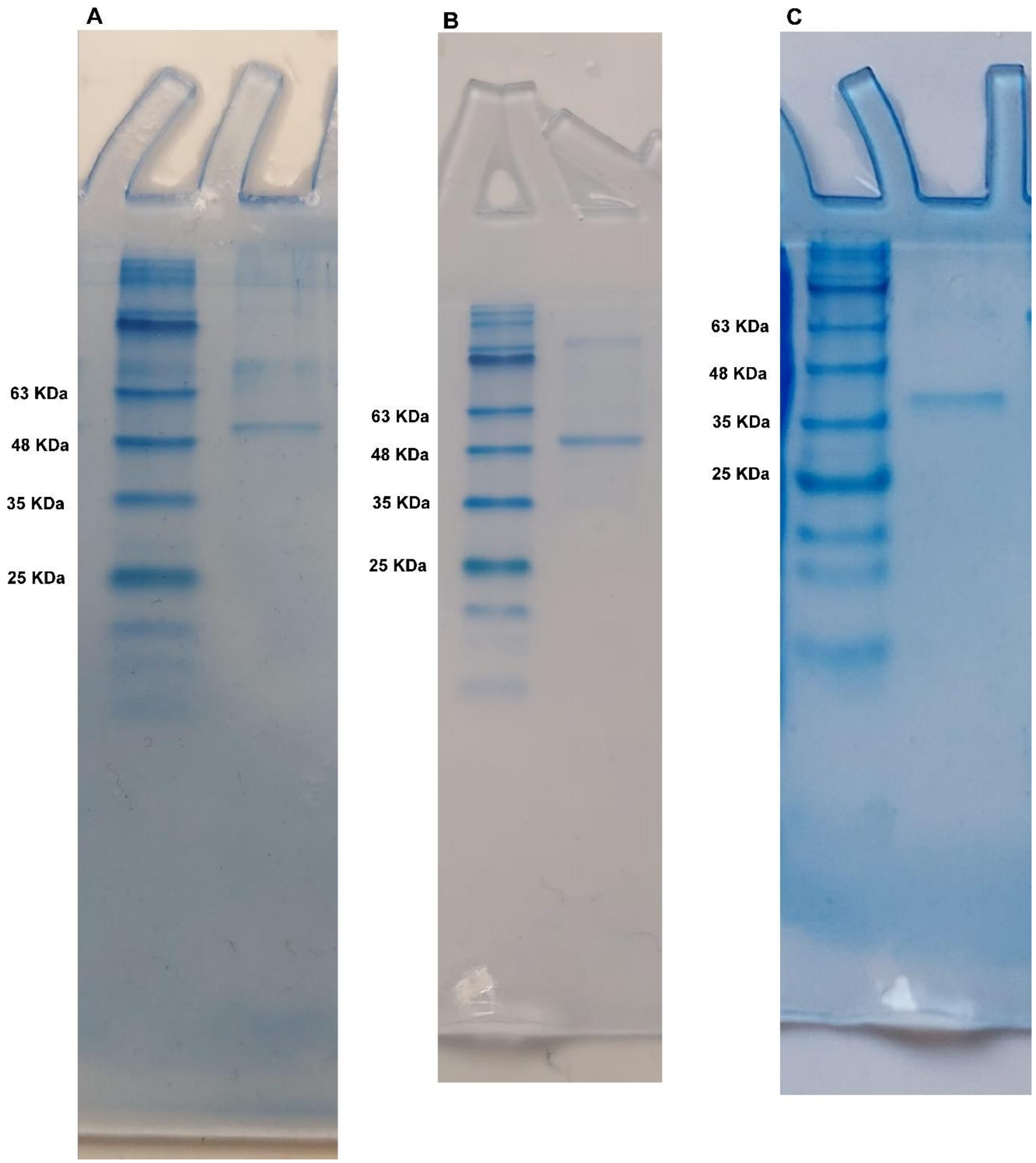

Supplementary Figure 4: Uncropped images of SDS PAGE gels for purified recombinant His-tagged proteins 6x-His-LY, 6x-His-KT and 6x-His-BD using Ni-NTA columns.

A photograph of coomassie blue-stained SDS-PAGE gel of Ni-NTA purified recombinant: (A) 6x-His-LY with a size of approximately 59.2 KDa, (B) 6x-His-KT with a size of approximately 57.9 KDa, (C) 6x-His-BD with a size of approximately 35.1 KDa.

Supplementary table 1: List of the top 104 putative protein candidates identified via bioinformatic analysis

Entry	Protein names	Gene names	Length in amino acid	localization (bsortb)	Homology to human proteom	Homology to mouse proteom	adhesion	Stability prediction (threshold ≤ 40)	MWT (kDa)	Antigenicity (VaxiJen threshold = 0.4)	Transmembrane helix	
					Vaxign			ProtParam			TMHMM TM prediction	HMMTOP TM prediction
B4EAX8	Family M23 peptidase	BCAL1893	299	OuterMembrane	no	no	0.654	38.58	30.64	0.5339	0	0
B4E8L2	Flagellar basal-body rod protein FlgG (Distal rod protein)	flgG BCAL0569	262	Extracellular	no	no	0.763	23.37	27.51	0.5942	0	0
B4EA18	Putative acylhydrolase	BCAL2834	378	OuterMembrane	no	no	0.719	24.78	39	0.4674	0	1
B4E786	Putative lipoprotein	BCAL2520	584	Extracellular	no	no	0.883	23.87	60.15	0.5147	0	1
B4EQ19	Putative lipoprotein	BCAS0251	385	Extracellular	no	no	0.89	30.81	38.67	0.7607	0	1
B4EP19	Putative lipoprotein	BCAS0625	267	Extracellular	no	no	0.778	36.56	27.32	0.9314	0	0
B4EBM9	Putative porin signal peptide protein	BCAL2002	348	OuterMembrane	no	no	0.655	30.71	36.69	0.5011	0	0
B4E8K9	Basal-body rod modification protein FlgD	flgD BCAL0566	234	Extracellular	no	no	0.693	18.5	23.19	0.6926	0	0
B4ENY7	Efflux system outer membrane protein	BCAS0593	475	OuterMembrane	no	no	0.622	35.64	51.3	0.4897	0	0
B4EF94	Exported lipase LipA (EC 3.1.1.3)	lipA BCAM0949	364	Extracellular	no	no	0.707	24.45	37.68	0.4937	1	1
B4EDI7	Flagellar hook-associated protein 2 (HAP2) (Flagellar cap	fliD1 BCAL0113	502	Extracellular	no	no	0.904	15.96	49.03	0.7275	0	0
B4E8L9	Flagellar hook-associated protein 3 (HAP3)	flgL BCAL0577	411	Extracellular	no	no	0.846	24.74	42.13	0.6630	0	0
B4EMF0	Hypothetical phage protein	BCAM1886	129	Extracellular	no	no	0.555	38.75	13.32	0.6434	0	0
B4EMH2	Hypothetical phage protein	BCAM1907	220	Extracellular	no	no	0.701	30.03	23.11	0.4323	0	1
B4EII1	Levansucrase (EC 2.4.1.10)	BCAM1405	520	Extracellular	no	no	0.806	30.82	55.92	0.5606	0	1
B4E8K5	Negative regulator of flagellin synthesis (Anti-sigma-28 factor)	flgM BCAL0562	108	Extracellular	no	no	0.686	32.1	10.67	0.8427	0	0
B4EHR2	Opacity family porin protein	BCAM0231	238	OuterMembrane	no	no	0.849	16.22	25.71	0.5835	0	1
B4E6X4	Phosphoesterase family protein	BCAL1409	528	Extracellular	no	no	0.688	32.09	56.09	0.4251	0	0
B4EGE7	Putative copper resistance protein C	BCAM2215	121	Extracellular	no	no	0.675	23.3	12.7	0.6295	1	0
B4EHY7	Putative cytochrome c	BCAM2369	398	Extracellular	no	no	0.698	35.09	41.63	0.5983	1	0
B4E8A9	Putative exported outer membrane porin protein	BCAL2615	362	OuterMembrane	no	no	0.931	20.75	38.22	0.6883	0	1
B4E6D9	Putative exported protein	BCAL2413	126	OuterMembrane	no	no	0.639	22.19	13.78	0.6376	0	0
B4EN73	Putative exported protein	BCAM2027a	196	Extracellular	no	no	0.893	20.11	17.73	1.1973	0	1
B4EPD8	Putative exported protein	BCAS0750	180	Extracellular	no	no	0.762	1.57	15.92	1.3450	0	1
B4E6M2	Putative exported protein	BCAL3492	127	Extracellular	no	no	0.681	32.72	12.23	1.605	1	0
B4EHH7	Putative exported protein with coagulation factor 5/8 type C-terminal domain	BCAM2328	469	Extracellular	no	no	0.949	25.58	49.54	0.585	1	1
B4ELI7	Putative gram-negative porin	BCAM2789	389	Outer Membrane	no	no	0.753	25.95	40.25	0.7535	0	1
B4EM19	Putative gram-negative porin	BCAM0717	386	Outer Membrane	no	no	0.92	17.66	40.68	0.6007	0	0
B4EJX5	Putative gram-negative porin	BCAM0473	362	Outer Membrane	no	no	0.815	25.41	37.93	0.5392	0	1
B4EKW5	Putative gram-negative porin	BCAM2696	356	Outer Membrane	no	no	0.735	32.75	37.68	0.6402	1	1
B4EQ72	Putative membrane protein	BCAS0306	423	Extracellular	no	no	0.89	22.11	43.33	0.7199	1	1
B4E6K3	Putative outer membrane porin	BCAL3473	383	OuterMembrane	no	no	0.803	14.62	39.9	0.4846	0	1
B4EFS3	Putative outer membrane porin	BCAM2126	363	OuterMembrane	no	no	0.81	28.42	38.82	0.6283	0	0
B4EGK5	Putative outer membrane protein-OmpW family	BCAM0081	241	OuterMembrane	no	no	0.729	25.83	25.3	0.6779	0	1
B4ELB0	Putative porin	BCAM1732	367	OuterMembrane	no	no	0.915	38.42	39.75	0.6198	1	1
B4EMV6	Putative porin	BCAM1974	402	OuterMembrane	no	no	0.741	23.4	41.78	0.6354	0	0
B4EM59	Putative porin	BCAM0757	375	OuterMembrane	no	no	0.824	28.22	39.62	0.5184	0	0
B4EI78	Putative porin	opcI BCAM0267	344	Outer Membrane	no	no	0.819	17.94	36.08	0.5946	0	1
B4EJP1	Putative porin	BCAM1549	398	OuterMembrane	no	no	0.862	22.3	41.46	0.6975	0	1
B4ELL3	Putative porin	BCAM0663	352	OuterMembrane	no	no	0.8	37.45	36.8	0.6128	0	1
B4ELU8	Putative porin	BCAM1787	384	OuterMembrane	no	no	0.699	16.7	40.84	0.6167	1	1
B4EHS7	Putative porin	BCAM1287	395	OuterMembrane	no	no	0.891	22.58	40.62	0.6488	0	0
B4EQ24	Putative porin protein	BCAS0256	388	OuterMembrane	no	no	0.922	23.78	39.65	0.6968	1	1
B4ENK6	Putative porin protein	BCAS0460	371	OuterMembrane	no	no	0.811	16.6	38.11	0.5074	0	0
B4E8U9	Putative type-1 fimbrial protein	BCAL1677	170	Extracellular	no	no	0.788	11.66	17.35	0.7156	0	1
B4E6R5	Putative type VI secretion system protein TssD	BCAL0343	167	extracellular	no	no	0.667	39.48	18.42	0.9809	0	0
B4EIX4	Efflux system outer membrane protein	BCAM1419	501	OuterMembrane	no	no	0.91	38.58	52.96	0.576	0	1
B4EFJ6	Flagellar hook protein FlgE	flgE2 BCAM0987	414	Extracellular	no	no	0.854	19.46	42.59	0.7054	0	0
B4E8L0	Flagellar hook protein FlgE	flgE1 BCAL0567	413	Extracellular	no	no	0.867	17.59	42.51	0.6924	0	0
B4EPM1	[illegible] associated protein 2 (HAP2) (Flagellar cap protein)	fliD2 BCAS0104	504	Extracellular	no	no	0.884	11.08	50.08	0.7376	0	0
B4EDI8	Flagellin	fliC BCAL0114	384	Extracellular	no	no	0.793	28.3	38.79	0.6922	0	0
B4E824	Flp type pilus assembly protein	BCAL1528	443	Outer Membrane	no	no	0.661	30.02	45.67	0.5531	0	0
B4ELG0	Giant cable pilus	cblA BCAM2761	166	Extracellular	no	no	0.929	20.74	17.05	0.6868	0	0
B4EPF2	Multidrug efflux system outer membrane protein	oprB BCAS0764	500	Outer Membrane	no	no	0.536	39.89	53.59	0.6012	1	1
B4E6M5	Outer membrane porin lipoprotein	BCAL3495	375	OuterMembrane	no	no	0.687	14.54	40.75	0.5308	0	1
B4EAW6	Outer membrane protein assembly factor BamB	bamB BCAL1881	381	OuterMembrane	no	no	0.608	33.39	40	0.5693	0	1
B4EJ27	Phosphoesterase family protein	BCAM1474	557	Extracellular	no	no	0.875	28.58	58.38	0.6678	0	1
B4EFD1	Porin	BCAM2063	495	OuterMembrane	no	no	0.806	36.75	52	0.6467	0	1
B4EQG4	Putative conjugative transfer protein	pBCA027	414	OuterMembrane	no	no	0.763	29.19	44.17	0.6534	1	1
B4E7M5	Putative exported heme utilisation related protein	BCAL1522	561	OuterMembrane	no	no	0.675	35.31	60.65	0.6535	0	0
B4EG68	Putative exported phage protein	BCAM1087	332	Extracellular	no	no	0.749	30.03	33.98	0.4445	0	1
B4EG70	Putative exported phage protein	BCAM1089	518	Extracellular	no	no	0.923	36.22	54.1	0.6182	0	1
B4EAC5	Putative exported protein	BCAL1826	221	Extracellular	no	no	0.844	20.04	22.59	0.7214	0	0
B4EKU7	Putative exported protein	BCAM2678	548	Extracellular	no	no	0.702	20.64	57.06	0.6074	0	0
B4EJE8	Putative exported protein	BCAM0425	532	Extracellular	no	no	0.851	23.12	56.31	0.6814	0	0
B4ED12	Putative exported protein	BCAL1076	362	OuterMembrane	no	no	0.68	30.73	37.15	0.5177	0	1
B4E811	Putative flagellar hook-length control protein FliK	fliK BCAL0520	452	Extracellular	no	no	0.759	33.79	43.53	0.8540	0	0
B4EJT3	Putative gram-negative porin	BCAM2584	383	Outer Membrane	no	no	0.889	26.9	40.14	0.7234	0	1
B4EIM8	Putative gram-negative porin	BCAM2462	370	Outer Membrane	no	no	0.715	26.12	40.35	0.4408	0	1
B4EIL2	Putative gram-negative porin	BCAM2446	397	Outer Membrane	no	no	0.885	28.96	41.46	0.6729	0	1
B4E7M7	Putative lipoprotein	BCAL1524	557	Extracellular	no	no	0.904	12.19	49.28	0.9966	0	1
B4EEC6	Putative lipoprotein	BCAL0200	476	Extracellular	no	no	0.915	28.79	47.56	0.7576	0	1
B4E5S5	Putative lipoprotein	BCAL2397	383	Extracellular	no	no	0.835	13.98	33.85	0.9463	1	1
B4EL74	Putative lipoprotein	BCAM1696	301	Extracellular	no	no	0.942	36.43	29.72	0.7299	0	1
B4EJA5	Putative lipoprotein	BCAM0384	335	Extracellular	no	no	0.919	25.12	34.26	1.0521	0	0
B4EJM5	Putative lipoprotein	BCAM1535	523	Extracellular	no	no	0.855	37.44	53.84	0.4263	0	1
B4ELF8	Putative minor pilin and initiator	cblD BCAM2759	387	Extracellular	no	no	0.543	35.08	41.81	0.6296	0	1
B4EB48	Putative ompA family protein	ompA BCAL2958	222	OuterMembrane	no	no	0.581	35.59	23.97	0.487	0	1
B4EHZ1	Putative outer membrane porin	BCAM2373	356	OuterMembrane	no	no	0.917	16.13	36.59	0.5857	0	1
B4EDN4	Putative outer membrane porin	BCAL1154	364	OuterMembrane	no	no	0.79	25.35	37.88	0.5071	0	0
B4EKZ2	Putative outer membrane porin protein	BCAM2723	490	OuterMembrane	no	no	0.804	19.39	52.56	0.5854	0	1
B4EHG0	Putative outer membrane porin protein	BCAM2311	379	OuterMembrane	no	no	0.791	17.21	40.52	0.5398	0	1
B4E947	Putative outer membrane porin protein	BCAL0624	358	OuterMembrane	no	no	0.77	33.69	38.33	0.5245	0	1
B4EG19	Putative outer membrane protein	BCAM0033	363	OuterMembrane	no	no	0.682	17.39	37.79	0.5230	1	1
B4E610	Putative outer membrane protein	BCAL0287	243	OuterMembrane	no	no	0.778	23.96	26.03	0.5425	1	1
B4EQ03	Putative outer membrane protein	BCAS0237	220	OuterMembrane	no	no	0.521	31.43	23.18	0.7826	0	0
B4E7B4	Putative outer membrane-bound lytic murein transglycosylase (EC 3.2.1.-)	BCAL0403	384	OuterMembrane	no	no	0.535	27.92	41.27	0.4062	1	1
B4EE97	Putative plasmid conjugal transfer protein	BCAL0173	257	Extracellular	no	no	0.845	36.12	27.51	0.5274	1	1
B4EMB7	Putative porin	BCAM1855	359	OuterMembrane	no	no	0.893	21.46	37.92	0.4294	0	1
B4EIH4	Putative porin	BCAM1398	399	OuterMembrane	no	no	0.866	17.58	41.34	0.635	0	1
B4EPI3	Putative porin	BCAS0066	390	OuterMembrane	no	no	0.897	19.84	40.54	0.5293	0	1
B4EJC8	Putative porin	BCAM0407	387	OuterMembrane	no	no	0.818	25.24	40.21	0.7422	0	0
B4EMR4	Putative porin	BCAM1931	359	OuterMembrane	no	no	0.915	32.16	37.52	0.6868	0	1
B4EJ10	Putative porin	BCAM1455	380	OuterMembrane	no	no	0.879	17.29	39.88	0.6744	0	0
B4EIF2	Putative porin	BCAM1376	363	OuterMembrane	no	no	0.843	27.3	38.61	0.6435	0	1
B4E688	Putative porin	BCAL1368	386	OuterMembrane	no	no	0.914	25.88	40.32	0.679	0	1
B4EFM3	Putative porin	BCAM1015	386	OuterMembrane	no	no	0.89	17.54	39.62	0.6477	0	0
B4EEF8	Putative porin	BCAL1221	359	OuterMembrane	no	no	0.846	28.1	37.66	0.6621	0	1
B4ENG3	Putative porin protein	BCAS0415	394	OuterMembrane	no	no	0.859	23.59	40.9	0.7687	0	1
B4EPN8	Putative porin protein	BCAS0121	362	OuterMembrane	no	no	0.709	26.96	39.5	0.5524	0	1
B4E9D3	Putative porin protein	BCAL1734	404	OuterMembrane	no	no	0.835	31.18	42.32	0.5512	0	1
B4E5U1	Serine peptidase, family S10	BCAL3411	536	Extracellular	no	no	0.745	31.05	57.13	0.4921	0	0
B4EIP3	Serine peptidase, family S10	BCAM2477	558	Extracellular	no	no	0.719	36.36	60.12	0.6017	0	0
B4ENF7	Zinc metalloprotease ZmpA	zmpA BCAS0409	565	Extracellular	no	no	0.85	24.26	59.91	0.6513	0	1

Supplementary table 2: Number of IgG and IgA specific B-cell epitopes in the shortlisted 19 proteins candidates identified via IgPred

Entry	Protein names	Gene names	IgA	IgG
B4EG19	Putative outer membrane protein	BCAM0033	6	65
B4E688	Putative porin	BCAL1368	7	40
B4EEF8	Putative porin	BCAL1221	8	25
B4E8L0	Flagellar hook protein FlgE	flgE1 BCAL0567	8	30
B4ENF7	Zinc metalloprotease ZmpA	zmpA BCAS0409	8	61
B4EJA5	Putative lipoprotein	BCAM0384	9	27
B4EMR4	Putative porin	BCAM1931	10	33
B4EIH4	Putative porin	BCAM1398	10	45
B4EIM8	Putative gram-negative porin	BCAM2462	11	82
B4E9D3	Putative porin protein	BCAL1734	12	30
B4ENG3	Putative porin protein	BCAS0415	12	32
B4EAW6	Outer membrane protein assembly factor BamB	bamB BCAL1881	12	46
B4E7M7	Putative lipoprotein	BCAL1524	12	143
B4E7M5	Putative exported heme utilisation related protein	BCAL1522	13	63
B4E5S5	Putative lipoprotein	BCAL2397	14	33
B4EIP3	Serine peptidase, family S10	BCAM2477	14	70
B4EKU7	Putative exported protein	BCAM2678	20	47
B4EKZ2	Putative outer membrane porin protein	BCAM2723	21	90
B4EJ27	Phosphoesterase family protein	BCAM1474	22	39

Supplementary table 3: The number of predicted peptides binder to each of the tested MHC class I alleles in the shortlisted 19 protein candidates

Protein	B4EG19	B4E688	B4EEF8	B4E8L0	B4ENF7	B4EJA5	B4EMR4	B4EIH4	B4EIM8	B4E9D3	B4ENG3	B4EAW6	B4E7M7	B4E7M5	B4E5S5	B4EIP3	B4EKU7	B4EKZ2	B4EJ27
MHC I Alleles	Number of predicted peptides binders																		
A*01:01	9	20	13	11	23	10	15	18	15	16	17	6	0	16	0	21	16	17	21
A*02:01	9	10	11	13	15	6	6	8	13	11	9	7	11	18	11	17	22	8	10
A*02:03	15	11	12	14	17	12	10	15	15	14	11	11	19	23	19	21	23	14	10
A*02:06	16	12	15	20	27	9	9	25	18	18	15	11	20	25	19	22	31	16	19
A*03:01	5	5	9	2	3	4	9	10	8	12	5	6	4	13	2	16	12	8	10
A*11:01	8	8	9	9	6	5	11	14	8	13	11	7	3	16	3	19	11	9	14
A*23:01	7	9	10	6	7	7	8	17	15	11	12	8	0	15	1	16	8	13	9
A*24:02	9	9	12	7	8	8	8	17	14	12	12	8	0	15	1	14	8	12	9
A*26:01	22	27	23	19	20	12	21	26	19	15	14	11	10	24	5	28	20	19	24
A*30:01	14	8	10	3	21	5	11	17	19	12	11	8	5	25	2	21	25	17	18
A*30:02	15	25	20	15	21	18	21	24	24	19	22	6	0	19	0	29	15	26	20
A*31:01	10	8	9	2	14	2	9	5	8	7	5	4	1	11	0	12	9	2	3
A*32:01	18	16	20	14	16	5	18	26	20	14	21	8	10	27	8	27	18	25	18
A*33:01	6	6	9	3	16	2	10	3	10	7	4	5	1	13	0	9	7	3	6
A*68:01	6	7	8	4	11	8	12	4	8	10	7	3	1	20	1	12	8	7	8
A*68:02	11	17	7	26	19	12	7	22	9	14	16	6	41	29	36	28	28	16	22
B*07:02	7	1	6	7	14	9	5	4	6	1	4	13	18	16	12	13	15	9	11
B*08:01	10	4	7	4	11	3	3	9	11	11	10	9	6	23	2	15	17	16	9
B*15:01	17	22	21	16	16	8	24	23	22	18	23	11	3	15	3	24	10	26	18
B*35:01	20	25	23	24	23	17	25	24	21	24	17	18	9	20	8	27	26	29	28
B*40:01	1	2	7	2	9	1	2	3	4	3	3	10	0	7	1	6	3	9	5
B*44:02	5	4	10	1	14	5	6	4	6	5	2	9	0	10	0	8	4	11	10
B*44:03	3	4	9	0	13	4	6	3	5	3	3	6	0	9	0	6	4	11	9
B*51:01	8	10	7	18	15	10	3	6	7	10	5	18	14	19	8	17	17	12	20
B*53:01	13	13	16	16	13	16	15	18	13	10	10	15	7	16	2	20	16	18	20
B*57:01	8	8	11	12	8	3	13	16	9	5	12	11	0	16	0	10	8	16	8
B*58:01	7	16	15	18	8	4	19	21	12	7	12	13	2	25	1	16	8	20	11

Supplementary Table 4: The number of predicted peptides binder to each of the tested MHC class II alleles in the shortlisted 17 protein candidates																	
**Protein**	**B4EG19**	**B4E688**	**B4EEF8**	**B4E8L0**	**B4ENF7**	**B4EJA5**	**B4EMR4**	**B4EIH4**	**B4EIM8**	**B4E9D3**	**B4ENG3**	**B4EAW6**	**B4E7M5**	**B4EIP3**	**B4EKU7**	**B4EKZ2**	**B4EJ27**
**MHC II Alleles**	**Number of predicted peptides binder**																
DRB1*01:01	12	12	13	16	11	15	9	15	12	10	18	14	23	21	13	12	13
DRB1*03:01	5	3	3	11	11	4	7	2	6	4	3	8	9	8	14	5	7
DRB1*04:01	13	13	13	24	21	17	14	21	14	16	17	16	16	20	21	19	20
DRB1*04:05	9	10	9	19	14	14	11	14	10	15	16	12	16	16	16	15	17
DRB1*07:01	10	14	7	16	14	9	12	15	10	12	17	12	21	15	14	11	18
DRB1*08:02	9	1	10	9	15	10	14	14	11	14	10	13	14	17	13	16	16
DRB1*09:01	11	1	9	16	18	10	19	17	12	16	18	11	19	16	14	14	16
DRB1*11:01	7	7	6	6	12	11	12	13	8	10	12	12	11	11	12	10	14
DRB1*12:01	4	4	4	4	9	2	4	4	8	10	3	7	21	7	7	8	5
DRB1*13:02	6	6	5	15	10	7	11	6	11	5	10	6	17	9	9	10	11
DRB1*15:01	4	6	8	13	11	6	8	9	8	8	14	8	20	13	7	13	11
DRB3*01:01	7	6	10	18	18	10	10	8	7	10	8	10	16	13	12	13	14
DRB3*02:02	11	11	11	21	25	18	16	20	14	15	18	12	23	16	21	21	14
DRB4*01:01	4	4	3	12	17	6	7	4	6	9	11	10	22	18	12	9	9
DRB5*01:01	8	12	10	12	9	10	12	14	13	14	16	8	16	16	10	14	11
DPA1*01/DPB1*04:01	0	0	0	0	0	0	0	0	0	0	0	0	0	0	0	0	0
DPA1*01:03/DPB1*02:01	9	15	12	9	12	7	13	14	14	13	8	8	17	22	12	20	13
DPA1*02:01/DPB1*01:01	6	16	9	11	9	10	11	10	13	10	11	7	1	14	15	13	12
DPA1*02:01/DPB1*05:01	3	11	6	7	9	8	10	6	11	8	11	4	17	11	14	11	8
DPA1*03:01/DPB1*04:02	8	14	9	9	7	10	11	8	16	12	10	8	21	14	16	17	14
DQA1*01:01/DQB1*05:01	7	6	8	10	14	8	5	2	9	4	10	6	14	17	5	11	12
DQA1*01:02/DQB1*06:02	13	0	10	19	23	9	18	12	7	11	18	11	17	11	15	17	15
DQA1*03:01/DQB1*03:02	4	7	6	9	13	11	4	5	9	4	9	8	16	15	13	13	14
DQA1*04:01/DQB1*04:02	11	18	8	15	23	19	12	14	11	15	17	12	16	23	14	19	18
DQA1*05:01/DQB1*02:01	3	8	3	15	13	12	4	9	8	7	8	6	21	20	13	8	11
DQA1*05:01/DQB1*03:01	19	21	10	18	24	15	17	19	9	9	17	16	19	20	16	17	20

Supplementary Table 5: Conservation of the 16 shortlisted candidates in the common BCC species with results recoreded as the number of hits with percentage identity and coverage of 80% obtained using the blastP tool of the NCBI non redundant database							
**Bcc strains**	***B. cenocepacia***	***B. multivorans***	**B. contaminans**	***B. cepacia***	***B. stabilis***	***B. vietnamiensis***	***B. dolosa***
**protein entry**	**Number of hits with percentage identity and coverage of 80% using the blastP tool of the NCBI non redundant databse**						
B4EG19	83	0	30	82	8	1	0
B4EEF8	83	0	34	96	8	2	0
B4E8L0	100	43	36	74	11	15	1
B4ENF7	71	0	21	55	9	1	0
B4EJA5	98	0	14	54	6	0	0
B4EMR4	22	19	11	30	4	8	2
B4EIH4	23	41	20	32	6	15	6
B4EIM8	40	0	21	46	7	1	0
B4E9D3	27	0	18	54	11	0	0
B4ENG3	55	0	22	52	8	1	0
B4EAW6	24	29	7	20	6	10	3
B4E7M5	45	36	22	50	7	24	4
B4EIP3	53	49	19	57	7	33	3
B4EKU7	91	100	31	100	10	45	0
B4EKZ2	47	59	12	31	8	28	7
B4EJ27	41	54	20	69	7	19	5

Supplementary table 6. List of the major histocompatibility class I & II alleles used in the TepiTool predictions.

Class	Alleles (human)
**MHC I alleles**	A*01:01, A*02:01, A*02:03, A*02:06, A*03:01, A*11:01, A*23:01, A*24:02, A*26:01, A*30:01, A*30:02, A*31:01, A*32:01, A*33:01, A*68:01, A*68:02, B*07:02, B*08:01, B*15:01, B*35:01, B*40:01, B*44:02, B*44:03, B*51:01, B*53:01, B*57:01, B*58:01
**MHC II alleles**	DRB1*01:01, DRB1*03:01, DRB1*04:01, DRB1*04:05, DRB1*07:01, DRB1*08:02, DRB1*09:01, DRB1*11:01, DRB1*12:01, DRB1*13:02, DRB1*15:01, DRB3*01:01, DRB3*02:02, DRB4*01:01, DRB5*01:01, DPA1*01/DPB1*04:01, DPA1*01:03/DPB1*02:01, DPA1*02:01/DPB1*01:01, DPA1*02:01/DPB1*05:01, DPA1*03:01/DPB1*04:02, DQA1*01:01/DQB1*05:01, DQA1*01:02/DQB1*06:02, DQA1*03:01/DQB1*03:02, DQA1*04:01/DQB1*04:02, DQA1*05:01/DQB1*02:01, DQA1*05:01/DQB1*03:01
